# Asymmetric Mannich Reaction of α-(2-Nitrophenylsulfenyl)imino Acetamide: A Cyclization-Driven Process

**DOI:** 10.3390/molecules31030449

**Published:** 2026-01-27

**Authors:** Tsubasa Inokuma, Maki Miyamoto, Kazuki Okada, Genki Nagai, Ken-ichi Yamada

**Affiliations:** 1 Graduate School of Pharmaceutical Sciences, Tokushima University, Shomachi, Tokushima 770-8505, Japan; 2 Research Cluster on “Key Material Development”, Tokushima University, Shomachi, Tokushima 770-8505, Japan; 3 Research Cluster on “Hybrid Modality Exploration”, Tokushima University, Shomachi, Tokushima 770-8505, Japan

**Keywords:** Mannich reaction, proline, thiourea, homoserine, organocatalysis, imino acetamide, peptide, cyclic hemiaminal

## Abstract

An enantioselective Mannich reaction of 2-(2-nitrophenylsulfenylimino)acetamide is described. Under the optimized conditions using proline, triethylamine, and diarylthiourea additives, the initially formed Mannich adduct undergoes irreversible cyclization to afford cyclic hemiaminal products in 21–58% yield, with diastereomeric ratios ranging from 53:47 to 83:17. Enantioselectivity reaches up to 97% ee. The presence of N–H functionality of the substrate is crucial for this cyclization; in its absence, the Mannich adduct undergoes facile decomposition. Subsequent reduction in this intermediate efficiently furnished the corresponding homoserine derivative.

## 1. Introduction

Peptides have attracted considerable attention as promising therapeutic candidates due to their broad spectrum of biological activities, which arise from the diversity of their amino acid sequences [1,2]. However, peptides composed of only proteinogenic amino acids are often susceptible to rapid degradation under physiological conditions [3]. The incorporation of noncanonical amino acids into peptide chains not only greatly increases structural diversity but also improves proteolytic stability, thereby augmenting the prospect of peptide-based drug discovery [4,5]. A notable example of a noncanonical amino acid is homoserine, a one-carbon homolog of serine. Homoserine has been identified in several peptide natural products, some of which exhibit significant biological activities, such as keanumycins and tolaasin [6,7]. Moreover, the structural importance of homoserine has been highlighted in designed linear peptides, where its side chain promotes intramolecular hydrogen bonding and conformational stabilization [8]. Substitution at the β-position of homoserine offers the potential to modulate backbone conformation and fine-tune intermolecular interactions, as β-branched residues are well known to exert a strong influence on peptide backbone geometry [9,10]. Derivatization from aspartic acid represents a straightforward route to homoserine frameworks; however, such approaches are limited in the accessible substitution patterns at the β-position [11]. Among the methodologies for constructing homoserine derivatives, the reactions of α-imino esters with enolizable aldehydes or ketones have been extensively studied using proline-type organocatalysts [12,13,14,15]. Subsequent reduction of the resulting adduct readily furnishes the desired homoserine derivatives. However, most reported protocols employ a 4-methoxyphenyl group as the *N*-protecting group, the removal of which requires harsh conditions, thereby restricting the utility of these methods in the synthesis of peptides containing homoserine derivatives.

As a part of our studies on the asymmetric synthesis of noncanonical amino acids, we have developed asymmetric nucleophilic addition of indoles or arylboronates to *N*-2-nitrophenylsulfenyl (Nps)-protected α-imino carboxylic acid derivatives [16,17,18,19,20]. This strategy for constructing noncanonical amino acids enables the generation of a wide array of amino acid structures with diverse side chains by varying the nucleophiles. Furthermore, the Nps group can be removed under mild conditions, providing broad functional group tolerance and rendering this method suitable for the synthesis of peptides containing noncanonical amino acids [16,18]. We envisioned that extending the nucleophile scope to include enolizable aldehydes would broaden the synthetic versatility of this approach, enabling the preparation of homoserine derivatives. Herein, we report an asymmetric Mannich reaction using *N*-Nps iminoacetamide as a model substrate for *N*-Nps imino peptides.

## 2. Results and Discussion

A dichloromethane solution of Nps-iminoacetamide **1** and hexanal (**2a**; 1.5 equiv) was stirred at room temperature for 18 h in the presence of L-proline (**3**; 25 mol%). However, the desired adduct **5** was not obtained (Table 1, entry 1). Remarkably, when the reaction was conducted with triethylamine as an additive [21,22], a trace amount of hemiaminals **6a** and *epi*-**6a**, presumably arising from cyclization of **5**, was obtained (entry 2). Proline-catalyzed reactions are known to be enhanced by the use of hydrogen-bond-donor co-catalysts, such as thiourea derivatives [23,24,25]. The combined use of thiourea **4** and triethylamine improved the combined yield of **6a** and *epi*-**6a** to 24% (entry 3). The enantiomeric excess (ee) of **6a** was determined to be 92% by chiral stationary-phase HPLC analysis. Although the ee of *epi*-**6a** was also measured, the values proved unreliable—varying over repeated analyses due to the limited amount of available material—and therefore are not reported herein. Substituting triethylamine with 1,8-diazabicyclo[5.4.0]-undec-7-ene (DBU) led to sluggish conversion, giving only trace amounts of the hemiaminals (entry 4). Use of the guanidine-type base 1,5,7-triazabicyclo[4.4.0]dec-5-ene (TBD) gave the hemiaminals in slightly lower yield (18%) compared to triethylamine (entry 5). The use of the inorganic base Cs_2_CO_3_ decreased the yield, likely due to its limited solubility (entry 8). Increasing the loadings of **3**, **4**, and triethylamine led to enhanced formation of unidentified byproducts, presumably resulting from side reactions between the aldehyde and an Nps-amine generated via hydrolysis of **1**, without improving the hemiaminal yields (entry 9). Lowering the reaction temperature to 0 °C effectively suppressed side reactions, resulting in combined yield of 56% for the hemiaminals (entry 10), although catalytic amounts of **3**, **4**, and triethylamine gave poor yields (entry 11). Increasing the amount of the reagents likewise resulted in a diminished outcome (entry 12). When the reaction was conducted in polar solvents such as DMSO or DMF, no reaction occurred (entries 6 and 7). Among the solvents tested, chloroform gave the highest product yields (entries 6, 7, and 13–15).

Other aldehydes were examined as nucleophiles (Table 2). Although the yields and diastereoselectivity were lower than those obtained with **2a**, the reactions of branched aldehyde **2b** afforded the corresponding product with excellent enantioselectivity (entry 2). This observation indicates that steric hindrance arising from the R group in the aldehydes plays an important role in enantioinduction in this reaction. Phenyl-substituted aldehyde **2c** and aldehyde **2d**, featuring a fatty acid-like moiety, were also tolerated (entries 3 and 4). Furthermore, an amino group protected as a carbamate proved compatible under the reaction conditions (entry 5).

**Scheme 1:**
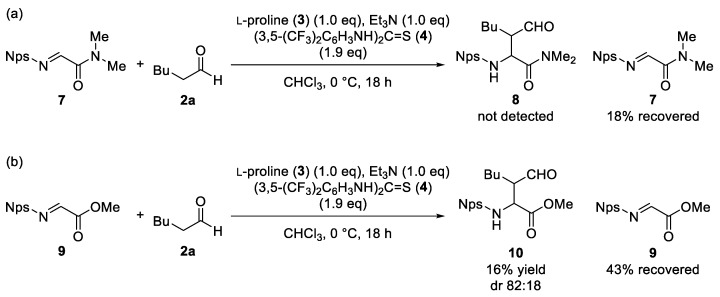
(**a**,**b**) The importance of the amide N–H moiety.

To gain insight into the reaction mechanism, control experiments were conducted (Scheme 1). The reaction of tertiary amide **7** under the optimized conditions failed to produce the corresponding adduct **8**, with **7** recovered in 18% yield (Scheme 1a). Imino ester **9**, expected to be more electrophilic than the corresponding amide **1**, gave only a trace amount of adduct **10** along with recovered **9** (43%). These results indicate that the subsequent cyclization step, which requires the presence of the amide N–H moiety, is crucial for the progress of the reaction [26].

**Scheme 2:**
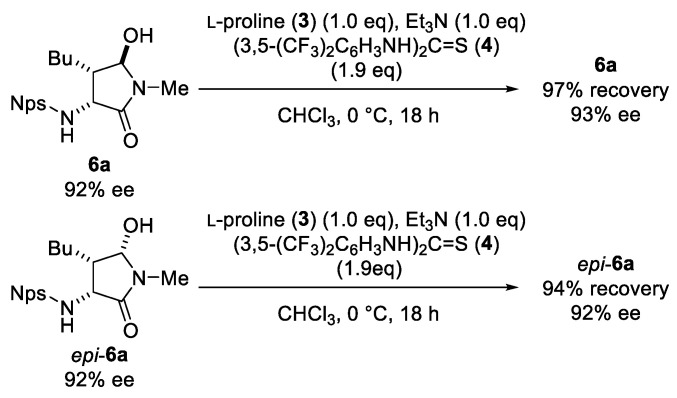
Irreversibility of the cyclization.

Next, the reversibility of the cyclization step was investigated (Scheme 2). When **6a** and *epi*-**6a** were separately subjected to the optimized Mannich reaction conditions, both compounds were recovered quantitatively without erosion of enantiomeric purity. These results suggest that the cyclization step proceeds irreversibly, stabilizing the otherwise labile intermediate **5** through its conversion into the more stable products **6a** and *epi*-**6a**.

**Scheme 3:**
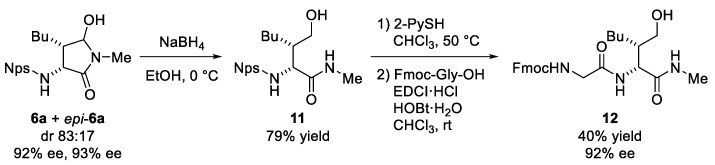
Synthesis of dipeptide **12** bearing homoserine derivative.

Finally, the conversion of the products into a homoserine-containing peptide was explored (Scheme 3). Reduction of the mixture with sodium borohydride in ethanol successfully afforded the homoserine derivative **11** as a sole diastereomer. The Nps group of **11** was then removed using 2-mercaptopyridine (2-PySH), and subsequent condensation with Fmoc-protected glycine yielded homoserine-containing dipeptide **12**. Throughout these transformations, the enantiomeric purity was fully preserved.

**Scheme 4:**
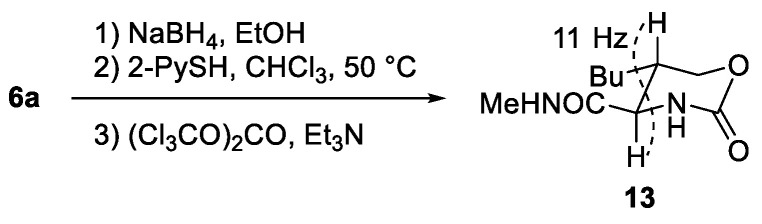
Determination of relative configuration of **6a**.

The 3,4-*cis* configuration of **6a** was established after its conversion to cyclic carbamate **13** through sodium borohydride reduction, Nps removal, and subsequent cyclization with triphosgene, on the basis of the large coupling constant (11 Hz) observed between the methine protons (Scheme 4). The relative configuration of the hemiaminal moieties was determined via nuclear Overhauser effect spectroscopy (NOESY), which revealed a clear correlation between the hemiaminal methine proton and a proton on the butyl side chain in **6a**, whereas no such correlation was observed in *epi*-**6a** (See Supplementary Materials). These observations indicate (3R*,4S*,5R*) and (3R*,4S*,5S*) configurations for **6a** and *epi*-**6a**, respectively. This assignment is further supported by DP4 analysis [27] comparing the experimental and calculated ^13^C NMR data (Table 3).

**Figure 1:**
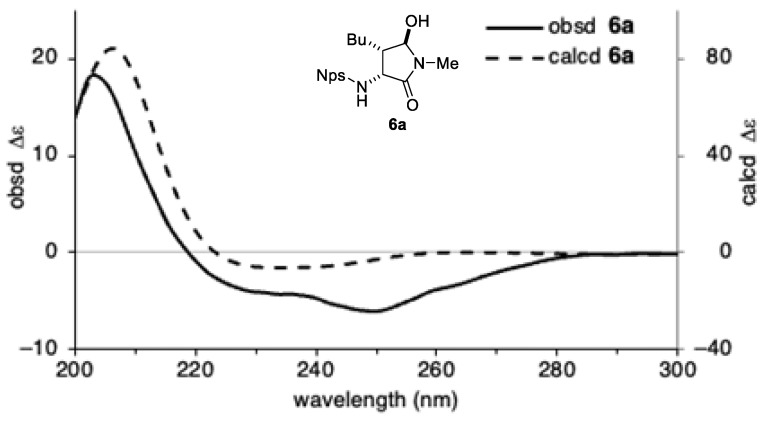
Experimentally observed and theoretically calculated ECD spectra of **6a**. The intensity of the vertical axis (Δε) in the observed spectrum (left) is scaled to 25% of that of the calculated spectrum (right).

The absolute configuration of **6a** was assigned by comparison of its experimental electronic circular dichroism (ECD) spectrum with spectra calculated by time-dependent density functional theory (TD-DFT) (Figure 1) [28,29]. Conformation analysis of **6a** was performed using the Merck Molecular Force Field (MMFF) to identify conformers with significant populations. The geometries of the resulting conformers were subsequently optimized at the B3LYP-D3/6-31G* theoretical level, incorporating solvent effects for methanol via the polarizable continuum model (PCM). ECD spectra were then computed at the TD-CAM-B3LYP/6-31+G* theoretical level with PCM solvation. Comparison of the experimentally recorded ECD spectrum of **6a** in methanol with the Bolzmann-weighted average of the calculated spectra led to the assignment of the absolute configuration of **6a** as 3*R*,4*S*,5*R*. The relative and absolute configurations of the other products were tentatively assigned by analogy.

## 3. Materials and Methods

### 3.1. General Information

All anhydrous reactions were carried out under a positive atmosphere of argon in dried glassware. Dehydrated solvents were purchased for the reactions and used without further desiccation. Analytical thin-layer chromatography was performed on Merck TLC silica gel 60F254 silica gel plates. Visualization was accomplished with molibudenium phosphate, *p*-anisaldehyde, Hannessian’s cocktail or ninhydrin. Column chromatography was performed using Silica Gel 60N (particle size 0.040–0.050 mm) purchased from Kanto Chemical Co., Inc. (Tokyo, Japan). NMR spectra were recorded using a Bruker AV400N or JEOL JNM-ECZL500R (JEOL Ltd., Tokyo, Japan) in the stated solvents using tetramethylsilane as an internal standard. Chemical shifts were reported in parts per million (ppm) on the δ scale from an internal standard (NMR descriptions: s = singlet, d = doublet, t = triplet, m = multiplet, br = broad). Coupling constants, *J*, are reported in Hertz. Mass spectra were recorded on a Waters/Micromass SQD2, MICROMASS^®^ LCT PREMIERTM (ESI-TOF) (Waters Corp., Milford, MA, USA). Optical rotations were measured using a JASCO P-2200 polarimeter (JASCO Corp., Tokyo, Japan) (concentration in g·dL^−1^). IR was measured using a JEOL FT-IR 6200 (JASCO Corp., Tokyo, Japan). Melting point was determined on J-SCIENCE RFS-10 (J-Science Lab. Co. Ltd., Kyoto, Japan). High-performance liquid chromatography (HPLC) analyses were performed on a Shimadzu PDA Detector SPD-M20A (Shimadzu Corp., Kyoto, Japan) equipped with two LC-20AD pumps. Unless otherwise noted, reagents were used without further purification. ECD spectra was measured by a JASCO J-1500 (JASCO Corp., Tokyo, Japan) spectrophotometer. The imines **1** [16], **7** [20], **9** [30], thiourea **4** [31], and aldehyde **2e** [32] were prepared according to the literature procedures.

### 3.2. General Procedure for Mannich Reaction of N-Nps Imino Acetamide ***1***: (3R,4S,5R)-4-Butyl-5-hydroxy-1-methyl-3-(2-nitrophenylthioamino)pyrrolidin-2-one (***6a***) and (3R,4S,5S)-4-Butyl-5-hydroxy-1-methyl-3-(2-nitrophenylthioamino)pyrrolidin-2-one (epi-***6a***)

A solution of **3** (23 mg, 0.20 mmol), **4** (0.19 g, 0.38 mmol), and Et_3_N (20 mg, 0.20 mmol) in CHCl_3_ (2.0 mL) was stirred at 0 °C. After 5 min, freshly distilled **2a** (37 μL, 0.30 mmol) was added, and the mixture was stirred at 0 °C for 5 min. Then, **1** (47.9 mg, 0.200 mmol) was added, and the mixture was stirred at 0 °C. After 18 h, the mixture was purified by silica gel flash chromatography (CHCl_3_/acetone 9:1) to give an 83:17 mixture of **6a** with 92% ee and *epi*-**6a** (39.6 mg, 58% yield). The ratio of **6a** and *epi*-**6a** was determined by the integral area of ^1^H NMR signals at 4.89 and 5.00 ppm. The ee was determined by HPLC analysis (Daicel Chiralpak AD-H; hexane/*i*-PrOH 90:10; 1.0 mL/min; 254 nm; **6a**: 15.2 min *ent*-**6a**: 18.7 min). The diastereomers were separated by silica gel column chromatography (hexane/ethyl acetate 1:1) for characterization.

**6a**: Yellow oil. [α]^25^_D_ +78 (*c* 1.61, CHCl_3_). ^1^H NMR (500 MHz, CDCl_3_): δ 8.28 (dd, *J* = 8.0, 1.0 Hz, 1H), 8.10 (dd, *J* = 8.0, 1.0 Hz, 1H), 7.66 (td, *J* = 8.0, 1.0 Hz, 1H), 7.27 (td, *J* = 8.0, 1.0 Hz, 1H), 4.89 (s, 1H), 4.00 (dd, *J* = 7.0, 4.0 Hz, 1H), 3.34 (d, *J* = 4.0 Hz, 1H), 2.94 (s, 3H), 2.61 (br s, 1H), 2.38 (m, 1H), 1.75–1.59 (m, 2H), 1.44–1.32 (m, 4H), 0.93 (t, *J* = 7.0 Hz, 3H). ^13^C NMR (125 MHz, CDCl_3_): δ 173.2, 144.6, 142.8, 134.2, 126.0, 125.0, 124.3, 87.1, 60.9, 45.8, 29.2, 27.7, 26.3, 22.8, 14.0. LRMS (ESI) (*m/z*): 362 [M+Na]^+^. HRMS (ESI) (*m/z*): [M+H]^+^ calcd for C_15_H_22_N_3_O_4_S, 340.1331; found, 340.1344. IR (NaCl): 2954, 2932, 2862, 1682, 1591, 1563, 1510, 1335, 1305, 736 cm^−1^.

*epi*-**6a**: Yellow powder of mp. 111–116 °C. [α]^25^_D_ +10 (*c* 1.42, CHCl_3_). ^1^H NMR (500 MHz, CDCl_3_): δ 8.34 (dd, *J* = 8.5, 1.0 Hz, 1H), 8.26 (dd, *J* = 8.5, 1.0 Hz, 1H), 7.71 (td, *J* = 8.5, 1.0 Hz, 1H), 7.28 (td, *J* = 8.5, 1.0 Hz, 1H), 5.00 (dd, *J* = 5.0, 8.0 Hz, 1H), 3.54 (t, *J* = 7.0 Hz, 1H), 3.32 (d, *J* = 7.0 Hz, 1H), 3.30 (d, J = 8.0 Hz, 1H), 2.94 (s, 3H), 2.28 (dtd, *J* = 8.0, 7.0, 5.0 Hz, 1H), 1.78–1.65 (m, 2H), 1.48–1.38 (m, 4H), 0.95 (t, *J* = 7.0 Hz, 3H). ^13^C NMR (125 MHz, CDCl_3_): δ 173.4, 144.5, 142.4, 134.1, 125.6, 125.05, 125.02, 85.6, 63.2, 42.9, 29.6, 27.7, 23.9, 22.9, 14.0. LRMS (ESI) (*m/z*): 362 [M+Na]^+^. HRMS (ESI) (*m/z*): [M+H]^+^ calcd for C_15_H_22_N_3_O_4_S, 340.1331; found, 340.1334. IR (NaCl): 3335, 2953, 2929, 2859, 1674, 1591, 1563, 1509, 1336, 1303 cm^−1^.

(3*R*,4*S*,5*R*)-5-Hydroxy-4-isopropyl-1-methyl-3-(2-nitrophenylthioamino)pyrrolidin-2-one (**6b**) and (3*R*,4*S*,5*S*)-5-hydroxy-4-isopropyl-1-methyl-3-(2-nitrophenylthioamino)pyrrolidin-2-one (*epi*-**6b**):

A similar procedure to that described for **6a** and *epi*-**6a**, using **2b** (33 μL, 0.31 mmol) instead of **2a**, gave a 54:46 mixture of **6b** with 97% ee and *epi*-**6b** (14.1 mg, 22% yield). The ratio of **6b** and *epi*-**6b** was determined by the integral area of ^1^H NMR signals at 4.91 and 5.01 ppm. The ee was determined by HPLC analysis (Daicel Chiralpak AD-H; hexane/*i*-PrOH 92.5:7.5; 1.0 mL/min; 254 nm; **6b**: 23.4 min *ent*-**6b**: 29.1 min). The diastereomers were separated by silica gel column chromatography (hexane/ethyl acetate 1:2) for characterization.

**6b:** Pale yellow oil. [α]^25^_D_ +93 (*c* 0.26, CHCl_3_). ^1^H NMR (500 MHz, CDCl_3_): δ 8.31 (dd, *J* = 8.5, 1.5 Hz, 1H), 8.05 (dd, *J* = 8.5, 1.5 Hz, 1H), 7.69 (td, *J* = 8.5, 1.0 Hz, 1H), 7.30 (td, *J* = 8.5, 1.0 Hz, 1H), 4.91 (d, *J* = 4.5 Hz, 1H), 4.00 (dd, *J* = 8.0, 3.0 Hz, 1H), 3.46 (d, *J* = 3.0 Hz, 1H), 2.93 (s, 3H), 2.36 (dd, *J* = 8.0, 3.0 Hz, 1H), 2.26 (m, 1H), 2.23 (m, 1H), 1.10 (d, *J* = 7.0 Hz, 3H), 0.73 (d, *J* = 7.0 Hz, 3H). ^13^C NMR (125 MHz, CDCl_3_): δ 173.3, 144.3, 143.1, 134.3, 126.2, 125.1, 124.3, 84.3, 59.6, 50.4, 27.1, 25.1, 21.1, 17.4. LRMS (ESI) (*m/z*): 348 [M+Na]^+^. HRMS (ESI) (*m/z*): [M+H]^+^ calcd for C_14_H_20_N_3_O_4_S, 326.1175; found, 326.1167. IR (NaCl): 2969, 2900, 2831, 1673, 528, 444, 409 cm^−1^.

*epi*-**6b:** Pale yellow oil. [α]^25^_D_ +4 (*c* 0.31, CHCl_3_). ^1^H NMR (400 MHz, CDCl_3_): δ 8.29 (d, *J* = 8.0 Hz, 1H), 8.27 (d, *J* = 8.0 Hz, 1H), 7.71 (t, *J* = 8.0 Hz, 1H), 7.29 (t, *J* = 8.0 Hz, 1H), 5.01 (d, *J* = 6.5, 5.0 Hz, 1H), 3.62 (t, *J* = 7.2 Hz, 1H), 3.29 (m, 1H), 2.94 (s, 3H), 2.13 (m, 1H), 1.85 (m, 1H), 1.14 (d, *J* = 6.5 Hz, 3H), 1.11 (d, *J* = 6.5 Hz, 3H). ^13^C NMR (125 MHz, CDCl_3_): δ 173.6, 144.4, 142.5, 134.0, 125.6, 125.24, 125.16, 85.4, 62.6, 50.8, 27.6, 23.4, 21.4, 20.2. LRMS (ESI) (*m/z*): 348 [M+Na]^+^. HRMS (ESI) (*m/z*): [M+H]^+^ calcd for C_14_H_20_N_3_O_4_S, 326.1175; found, 326.1184. IR (NaCl): 3740, 3672, 3645, 3613, 2928, 769, 527, 4443, 409 cm^−1^.

(3*R*,4*S*,5*R*)-4-Benzyl-5-hydroxy-1-methyl-3-(2-nitrophenylthioamino)pyrrolidin-2-one (**6c**) and (3*R*,4*S*,5*S*)-4-Benzyl-5-hydroxy-1-methyl-3-(2-nitrophenylthioamino)pyrrolidin-2-one (*epi*-**6c**):

A similar procedure to that described for **6a** and *epi*-**6a**, using **2c** (40 μL, 0.38 mmol) instead of **2a**, gave an 82:18 mixture of **6c** with 89% ee and *epi*-**6c** (15.8 mg, 21% yield). The ratio of **6c** and *epi*-**6c** was determined by the integral area of ^1^H NMR signals at 4.80 and 4.85 ppm. The ee was determined by HPLC analysis (Daicel Chiralpak AD-H; hexane/*i*-PrOH 90:10; 1.0 mL/min; 254 nm; **6c**: 29.1 min *ent*-**6c**: 32.3 min). The diastereomers were separated by silica gel column chromatography (hexane/ethyl acetate 1:2) for characterization.

**6c**: Pale yellow oil. [α]^25^_D_ +63 (*c* 1.33, CHCl_3_). ^1^H NMR (500 MHz, CDCl_3_): δ 8.30 (d, *J* = 8.5 Hz, 1H), 8.05 (d, *J* = 8.5 Hz, 1H), 7.68 (t, *J* = 8.5 Hz, 1H), 7.34 (t, *J* = 7.5 Hz, 2H), 7.30 (t, *J* = 7.5 Hz, 1H), 7.26–7.19 (m, 3H), 4.79 (d, *J* = 3.5 Hz, 1H), 4.12 (dd, *J* = 7.0, 3.0 Hz, 1H), 3.50 (d, *J* = 3.0 Hz, 1H), 3.17 (dd, *J* = 14.5, 4.5 Hz, 1H), 2.91 (s, 3H), 2.81 (dddd, *J* = 12.0, 7.0, 4.5, 3.5 Hz, 1H), 2.47 (brs, 1H), 2.20 (dd, J = 14.5, 12.0 Hz, 1H). ^13^C NMR (100 MHz, CDCl_3_): δ 172.9, 144.2, 143.1, 138.7, 134.3, 128.9, 128.8, 126.6, 126.1, 125.1, 124.2, 85.8, 60.4, 46.9, 32.9, 27.8. LRMS (ESI) (*m/z*): 396 [M+Na]^+^. HRMS (ESI) (*m/z*): [M+H]^+^ calcd for C_18_H_20_N_3_O_4_S, 374.1175; found, 374.1153. IR (NaCl): 3653, 3029, 2936, 2867, 1680, 1503, 1333, 468, 458, 435 cm^−1^.

*epi*-**6c**: Pale yellow oil. [α]^25^_D_ +0.7 (*c* 0.26, CHCl_3_). ^1^H NMR (400 MHz, CDCl_3_): δ 8.34 (dd, *J* = 8.0, 1.2 Hz, 1H), 8.30 (dd, *J* = 8.0, 1.2 Hz, 1H), 7.71 (t, *J* = 8.0 Hz, 1H), 7.38–7.24 (m, 6H), 4.85 (dd, *J* = 6.8, 4.8 Hz, 1H), 3.65 (t, *J* = 7.2 Hz, 1H), 3.27 (d, *J* = 7.2 Hz, 1H), 3.20–3.09 (m, 2H), 2.91 (s, 3H), 2.81 (m, 1H), 2.73 (m, 1H). ^13^C NMR (100 MHz, CDCl_3_): δ 172.9, 144.3, 142.6, 138.6, 134.2, 128.9, 128.8, 126.6, 125.7, 125.2, 125.0, 85.1, 63.2, 44.3, 30.4, 27.7. LRMS (ESI) (*m/z*): 396 [M+Na]^+^. HRMS (ESI) (*m/z*): [M+Na]^+^ calcd for C_18_H_19_N_3_NaO_4_S, 396.0994; found, 396.0988. IR (NaCl): 3653, 2969, 2936, 2862, 1501, 467, 460, 420 cm^−1^.

(3*R*,4*S*,5*R*,*Z*)-5-Hydroxy-1-methyl-3-(2-nitrophenylthioamino)-4-(oct-2-enyl)pyrrolidin-2-one (**6d**) and (3*R*,4*S*,5*S*,*Z*)-5-Hydroxy-1-methyl-3-(2-nitrophenylthioamino)-4-(oct-2-enyl)pyrrolidin-2-one (*epi*-**6d**):

A similar procedure to that described for **6a** and *epi*-**6a**, using **2d** (55 μL, 0.33 mmol) instead of **2a**, gave an 81:19 mixture of **6d** with 81% ee and *epi*-**6d** (17.6 mg, 22% yield). The ratio of **6d** and *epi*-**6d** was determined by the integral area of ^1^H NMR signals at 4.83 and 5.00 ppm. The ee was determined by HPLC analysis (COSMOSIL CHiRAL 3A; hexane/*i*-PrOH 90:10; 1.0 mL/min; 254 nm; **6d**: 10.8 min, *ent*-**6d**: 11.9 min). The diastereomers were separated by silica gel column chromatography (hexane/ethyl acetate 2:3) for characterization.

**6d**: Pale yellow oil. [α]^25^_D_ +36 (*c* 0.66, CHCl_3_). ^1^H NMR (500 MHz, CDCl_3_): δ 8.30 (dd, *J* = 8.5, 1.0 Hz, 1H), 8.07 (dd, *J* = 8.5, 1.0 Hz, 1H), 7.69 (td, *J* = 8.5, 1.0 Hz, 1H), 7.29 (td, *J* = 8.5, 1.0 Hz, 1H), 5.62 (m, 1H), 5.38 (m, 1H), 4.83 (s, 1H), 4.04 (dd, *J* = 7.0, 3.5 Hz, 1H), 3.42 (d, *J* = 3.5 Hz, 1H), 2.93 (s, 3H), 2.54–2.46 (m, 2H), 2.37 (dt, *J* = 15.0, 4.0 Hz, 1H), 2.05–1.90 (m, 3H), 1.40–1.24 (m, 7H), 0.89 (t, *J* = 7.0 Hz, 3H). ^13^C NMR (100 MHz, CDCl_3_): δ 173.0, 144.3, 143.1, 134.3, 133.4, 126.1, 125.5, 125.0, 124.3, 86.5, 60.3, 45.7, 31.5, 29.2, 27.7, 27.4, 24.8, 22.6, 14.0. LRMS (ESI) (*m/z*): 416 [M+Na]^+^. HRMS (ESI) (*m/z*): [M+H]^+^ calcd for C_19_H_28_N_3_O_4_S, 394.1801; found, 394.1815. IR (NaCl): 3561, 2927, 1679, 1509, 769, 527, 500, 445, 410 cm^−1^.

*epi*-**6d**: Pale yellow oil. [α]^25^_D_ −2 (*c* 0.27, CHCl_3_). ^1^H NMR (400 MHz, CDCl_3_): δ 8.33 (dd, *J* = 8.0, 0.8 Hz, 1H), 8.28 (dd, *J* = 8.0, 0.8 Hz, 1H), 7.72 (td, *J* = 8.0, 0.8 Hz, 1H), 7.30 (td, *J* = 8.0, 0.8 Hz, 1H), 5.54 (m, 1H), 5.46 (m, 1H), 5.01 (dd, *J* = 8.0, 4.8 Hz, 1H), 3.63 (t, *J* = 6.4 Hz, 1H), 3.26 (d, *J* = 6.4 Hz, 1H), 2.94 (s, 3H), 2.74 (m, 1H), 2.53–2.43 (m, 3H), 2.15–2.08 (m, 2H), 1.44–1.25 (m, 6H), 0.90 (t, *J* = 6.8 Hz, 3H). ^13^C NMR (125 MHz, CDCl_3_): δ 172.7, 144.3, 142.6, 134.2, 133.1, 125.73, 125.66, 125.1, 125.0, 85.2, 62.8, 42.8, 31.5, 29.7, 29.2, 27.5, 22.6, 22.3, 14.1. LRMS (ESI) (*m/z*): 416 [M+Na]^+^. HRMS (ESI) (*m/z*): [M+H]^+^ calcd for C_19_H_28_N_3_O_4_S, 394.1801; found, 394.1783. IR (NaCl): 3346, 3007, 2957, 2925, 2854, 1676, 1510, 1335, 1260 cm^−1^.

(3*R*,4*S*,5*R*)-4-(4-*tert*-Butoxycarbonylaminobutyl)-5-hydroxy-1-methyl-3-(2-nitrophenylthioamino)pyrrolidin-2-one (**6e**) and (3*R*,4*S*,5*S*)-4-(4-*tert*-Butoxycarbonylaminobutyl)-5-hydroxy-1-methyl-3-(2-nitrophenylthioamino)pyrrolidin-2-one (*epi*-**6e**):

A similar procedure to that described for **6a** and *epi*-**6a**, using **2e** (65 mg, 0.33 mmol) instead of **2a**, gave a 76:24 mixture of **6e** with 89% ee and *epi*-**6e** (25.6 mg, 28% yield). The ratio of **6e** and *epi*-**6e** was determined by the integral area of ^1^H NMR signals at 4.84 and 4.99 ppm. The ee was determined by HPLC analysis (COSMOSIL CHiRAL 3C; hexane/*i*-PrOH 80:20; 1.0 mL/min; 254 nm; **6e**: 24.1 min *ent*-**6e**: 18.5 min). The diastereomers were separated by preparative TLC (hexane/ethyl acetate 1:3) for characterization.

**6e**: Pale yellow oil. [α]^21^_D_ +38 (*c* 0.60, CHCl_3_). ^1^H NMR (400 MHz, CDCl_3_): δ 8.22 (dd, *J* = 8.4, 1.2 Hz, 1H), 8.03 (dd, *J* = 8.4, 1.2 Hz, 1H), 7.62 (td, *J* = 8.4, 1.2 Hz, 1H), 7.22 (td, *J* = 8.4, 1.2 Hz, 1H), 4.81 (s, 1H), 4.55 (br s, 1H), 3.94 (dd, *J* = 7.2, 3.6 Hz, 1H), 3.33 (d, *J* = 3.6 Hz, 1H), 3.06 (td, *J* = 9.4, 6.2 Hz, 2H), 2.86 (s, 3H), 2.31 (m, 1H), 1.70–1.42 (m, 6H), 1.36 (s, 9H). ^13^C NMR (125 MHz, CDCl_3_): δ 172.9, 156.3, 144.4, 143.0, 134.3, 126.1, 125.0, 124.4, 87.0, 79.4, 60.6, 45.6, 30.3, 28.42, 28.39, 28.35, 26.3, 24.0. HRMS (ESI) (*m/z*): [M+Na]^+^ calcd for C_20_H_30_N_4_NaO_6_S, 477.1784; found, 477.1800. IR (ATR): 3344, 3331, 2926, 2859, 1682, 1510, 1336, 1251 cm^−1^.

*epi*-**6e**: Pale yellow oil. [α]^22^_D_ +0.6 (*c* 0.17, CHCl_3_). ^1^H NMR (400 MHz, CDCl_3_): δ 8.43 (d, *J* = 8.0 Hz, 1H), 8.26 (d, *J* = 8.0 Hz, 1H), 7.73 (t, *J* = 8.0 Hz, 1H), 7.26 (t, *J* = 8.0 Hz, 1H), 4.99 (dd, *J* = 5.0, 4.0 Hz, 1H), 4.69 (m, 1H), 4.48 (m, 1H), 3.49–3.35 (m, 2H), 3.06 (m, 1H), 2.96 (s, 3H), 1.94–1.50 (m, 6H), 1.45 (s, 9H). ^13^C NMR (125 MHz, CDCl_3_): δ 173.5, 157.1, 145.5, 142.3, 134.2, 125.6, 125.1, 124.9, 85.0, 80.0, 64.8, 43.5, 38.8, 29.7, 29.2, 28.3, 27.8, 22.7. HRMS (ESI) (*m/z*): [M+Na]^+^ calcd for C_20_H_30_N_4_NaO_6_S, 477.1784; found, 477.1779. IR (ATR): 3391, 3319, 2926, 2854, 1683, 1512, 1337, 1252 cm^−1^.

### 3.3. Mannich Reaction of Imino Ester ***9*** (Scheme 1)

Methyl 3-Formyl-2-(2-nitrophenylthioamino)heptanoate (10)

A similar procedure to that described for **6a** and *epi*-**6a**, using **9** (48.0 mg, 0.200 mmol) instead of **1**, gave **10** (10.8 mg, 16% yield, 50:50 dr): [α]^25^_D_ +36 (*c* 0.66, CHCl_3_).

^1^H NMR (400 MHz, CDCl_3_): δ 9.75 (s, 0.5H), 9.67 (s, 0.5H), 8.29 (d, *J* = 8.0 Hz, 1H), 8.04 (d, *J* = 8.0 Hz, 0.5H), 7.90 (d, *J* = 8.0 Hz, 0.5H), 7.64 (t, *J* = 8.0 Hz, 1H), 7.28 (t, *J* = 8.0 Hz, 1H), 3.96 (dd, *J* = 8.4, 3.6 Hz, 0.5H), 3.82 (s, 1.5H), 3.81 (s, 1.5H), 3.80 (m, 0.5H), 3.64 (d, *J* = 8.4 Hz, 0.5H), 3.52 (d, *J* = 8.4 Hz, 0.5H), 2.94 (dd, *J* = 12.0, 7.0 Hz, 0.5H), 2.82 (dt, *J* = 8.4, 4.4 Hz, 0.5H), 1.99–1.78 (m, 1H), 1.73–1.52 (m, 1.5H), 1.45–1.26 (m, 3.5H), 0.91 (t, *J* = 6.8 Hz, 1.5H), 0.89 (t, J = 6.8 Hz, 1.5H). ^13^C NMR (100 MHz, CDCl_3_): δ 201.9, 201.5, 172.6, 144.6, 144.0, 142.62, 142.59, 133.9, 133.8, 125.8, 125.03, 124.95, 124.5, 124.4, 63.71, 63.69, 54.6, 54.4, 52.8, 30.9, 29.6, 29.5, 25.2, 24.7, 22.6, 22.5, 13.8. LRMS (ESI) (*m/z*): 341 [M+H]^+^. HRMS (ESI) (*m/z*): [M+H]^+^ calcd for C_15_H_21_N_2_O_5_S, 341.1171; found, 341.1162. IR (NaCl): 3340, 3096, 2955, 2929, 2859, 1728, 1592, 1564, 1511, 1336, 449 cm^−1^.

### 3.4. Synthesis of the Dipeptide ***12*** Bearing Homoserine Derivative (Scheme 3)

(2*R*,3*S*)-3-(Hydroxymethyl)-*N*-methyl-2-(2-nitrophenylthioamino)heptanamide (**11**)

To a solution of an 83:17 mixture of **6a** and *epi*-**6a** (88.5 mg, 0.261 mmol) in EtOH (7 mL), NaBH_4_ was added (49 mg, 1.3 mmol) at rt, and the mixture was stirred for 17 h. Then, the mixture was diluted with water and extracted with CH_2_Cl_2_. The organic layer was dried over Na_2_SO_4_, and evaporated to give **11** (69.7 mg, 79% yield) as a yellow solid with mp 149–155 °C: [α]^25^_D_ −195 (*c* 1.29, CHCl_3_).

^1^H NMR (500 MHz, CDCl_3_): δ 8.27 (dd, *J* = 8.5, 1.0 Hz, 1H), 7.87 (dd, *J* = 8.5, 1.0 Hz, 1H), 7.64 (td, *J* = 8.5, 1.0 Hz, 1H), 7.28 (td, *J* = 8.5, 1.0 Hz, 1H), 6.52 (br s, 1H), 3.93–3.85 (m, 2H), 3.70 (m, 1H), 3.54 (dd, *J* = 6.5, 4.0 Hz, 1H), 2.88 (d, *J* = 5.0 Hz, 3H), 2.62 (br s, 1H), 2.04 (m, 1H), 1.54 (m, 1H), 1.42–1.28 (m, 5H), 0.91 (t, *J* = 7.0 Hz, 3H). ^13^C NMR (125 MHz, CDCl_3_): δ 173.1, 144.8, 133.8, 126.0, 125.0, 124.2, 115.9, 67.2, 63.1, 44.0, 29.8, 27.0, 26.3, 22.9, 14.0. LRMS (ESI) (*m/z*): 364 [M+Na]^+^. HRMS (ESI) (*m/z*): [M+H]^+^ calcd for C_15_H_24_N_3_O_4_S, 342.1488; found, 342.1499. IR (NaCl): 3543, 3291, 3104, 2932, 2862, 1647, 1505, 1336, 1306, 731 cm^−1^.

(2*R*,3*S*)-2-(9*H*-Fluoren-9-yl)methyloxycarbonylamino-3-hydroxymethyl-*N*-methyl-heptanamide (**12**)

To a solution of **11** (58.9 mg, 0.173 mmol) in CHCl_3_ (2 mL), 2-mercaptopyridine was added (0.19 g, 1.7 mmol), and the mixture was stirred at rt for 1 h. The stirred mixture was heated in an oil bath at 50 °C for 5 h, concentrated in vacuo, and passed through silica gel (40 g) eluted with CHCl_3_/MeOH (5:1 to 2:1) to give a crude amine as a yellow oil (32 mg). To s solution of Fmoc-Gly-OH (48 mg, 0.16 mmol), and HOBt·H_2_O (32 mg, 0.24 mmol) in CHCl_3_ (2 mL), EDCI·HCl was added (46 mg, 0.24 mmol) and the crude amine in CHCl_3_ (1 mL), successively, at room temperature, and the mixture was stirred at the same temperature for 12 h. The mixture was diluted with CHCl_3_, washed with 1M HCl followed by H_2_O, dried over Na_2_SO_4_, evaporated in vacuo, and purified by silica gel column chromatography (EtOAc) to afford **12** with 92% ee (29.6 mg, 40% yield) as a white solid of mp. 188–198 °C: [α]^25^_D_ +36 (*c* 0.66, CHCl_3_). The ee was determined by HPLC analysis (Daicel Chiralpak AD-H; hexane/*i*-PrOH 80:20; 1.0 mL/min; 254 nm; **12**: 7.0 min, *ent*-**12**: 8.6 min)

^1^H NMR (500 MHz, DMSO-*d*_6_): δ 7.88 (d, *J* = 7.5 Hz, 2H), 7.86–7.80 (m, 2H), 7.70 (d, *J* = 7.5 Hz, 2H), 7.61 (t, *J* = 6.0 Hz, 1H), 7.41 (t, *J* = 7.5 Hz, 2H), 7.32 (t, *J* = 7.5 Hz, 2H), 4.56 (t, *J* = 4.5 Hz, 1H), 4.30–4.19 (m, 4H), 3.67 (dd, *J* = 17.0, 6.0 Hz, 1H), 3.64 (dd, *J* = 17.0, 6.0 Hz, 1H), 2.57 (d, *J* = 4.5 Hz, 1H), 1.77 (m, 1H), 1.38–1.13 (m, 6H), 0.82 (t, *J* = 6.5 Hz, 3H). ^13^C NMR (125 MHz, DMSO-*d*_6_): δ 171.5, 169.2, 156.6, 143.9, 140.7, 127.7, 127.2, 125.3, 120.2, 65.8, 60.3, 54.1, 46.6, 43.6, 41.6, 28.7, 27.1, 25.6, 22.4, 14.0. LRMS (ESI) (*m*/*z*): 468 [M+H]^+^. HRMS (ESI) (*m*/*z*): [M+H]^+^ calcd for C_26_H_34_N_3_O_5_, 468.2498; found, 468.2522. IR (KBr): 3653, 3065, 3019, 2924, 2852, 2359, 2331, 1711, 733, 483 cm^−1^.

### 3.5. Determination of Relative Configuration of ***6a*** (Scheme 4)

(4*R*,5*S*)-5-Butyl-*N*-methyl-2-oxo-1,3-oxazinane-4-carboxamide (**13**)

To a solution of **7** (17.5 mg, 51.3 μmol) in CHCl_3_ (1 mL) 2-mercaptopyridine was added (57 mg, 0.51 mmol). The resulting mixture was stirred at 50 °C for 5 h, concentrated in vacuo, and passed through silica gel (10 g) using CHCl_3_/MeOH (5:1 to 2:1) as the eluent to give a crude amine as a yellow oil (12.2 mg). To a solution of the crude amine and Et_3_N (72 μL, 0.51 mmol) in CH_2_Cl_2_ (1 mL) triphosgene was added (17 mg, 56 μmol) at 0 °C, and the resulting mixture was stirred at rt. After 30 min, the reaction was quenched by the addition of saturated aqueous NaHCO_3_, and was extracted with CH_2_Cl_2_. The aqueous later was further extracted with CH_2_Cl_2_ (twice), and the combined organic layers were dried over Na_2_SO_4_, and evaporated in vacuo to give a crude amine such as a yellow wax (20 mg).

^1^H NMR (500 MHz, CDCl_3_): δ 4.58 (dd, *J* = 11.5, 5.0 Hz, 1H), 4.35 (d, *J* = 11.0 Hz, 1H), 4.10 (dd, *J* = 11.0, 11.5 Hz, 1H), 3.17 (d, *J* = 2.0 Hz, 3H), 2.35–2.24 (m, 2H), 1.44–1.27 (m, 7H), 0.93 (t, *J* = 7.0 Hz, 3H).

### 3.6. Calculations of the ^13^C NMR Chemical Shifts (Table 3)

Conformational searches for each diastereomer were conducted using the MMFF in Spartan 18 program [33]. Conformers exhibiting relative energies greater than 15 kJ/mol above the lowest energy conformer, as determined by single-point energy calculations at the ωB97X-D/6-31G* theoretical level, were discarded. The remaining conformers were fully optimized at the ωB97X-D/6-31G* level and subjected to ^13^C NMR calculations at the same theoretical level. The computed chemical shifts were empirically corrected using the Spartan 18 program and averaged according to Bolzmann populations derived from single-point energy calculations at the ωB97X-V/6-311+G(2df,2p) theoretical level.

### 3.7. Calculations of the ECD Spectra (Figure 1)

Conformational searches of **6a** were performed using the MMFF in Spartan 18 program [33]. Conformers with relative energies exceeding 15 kJ/mol above the most stable geometry, as determined by single-point energy calculations at the B3LYP-D3/6-31G*/PCM(MeOH) theoretical level, were excluded. The remaining conformers were fully optimized at the B3LYP-D3/6-31G*/PCM(MeOH) level using Gaussian 16 program [34]. The optimized geometries were subjected to TD-DFT calculations at the TD-CAM-B3LYP/6-31+G*/PCM(MeOH) theoretical level. The resulting rotatory strengths for the lowest 25 excited states were converted into Gaussian-type bands with a half-width of 0.25 eV. The calculated ECD spectrum was uniformly red-shifted by 20 nm.

## 4. Conclusions

We have developed an asymmetric Mannich reaction of an α-Nps-imino amide with aldehyde nucleophiles. The reaction was effectively facilitated by a cooperative system composing proline, thiourea, and triethylamine. Mechanistic studies revealed that the irreversible cyclization involving the amide N–H moiety and the formyl group is crucial for suppressing undesired side reactions. The resulting cyclic hemiaminals were readily converted into a peptide incorporating a homoserine derivative. Further optimization and applications of this methodology are currently underway.

## Figures and Tables

**Table 1:** Optimization of the Mannich reaction of *N*-Nps iminoacetamide^[a]^.

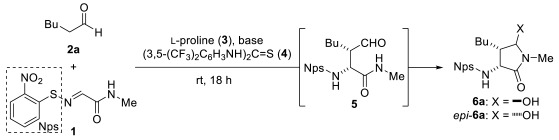							
Entry	Base (equiv)	3 (equiv)	4 (equiv)	Solvent	Yield (%)^[b]^	dr^[c]^	ee of 6a (%)^[d]^
1	---	0.25	---	CH_2_Cl_2_	---	---	---
2	Et_3_N (0.25)	0.25	---	CH_2_Cl_2_	Trace	---	---
3	Et_3_N (0.25)	0.25	0.50	CH_2_Cl_2_	24	81:19	92
4	DBU (0.25)	0.25	0.50	CH_2_Cl_2_	Trace	---	---
5	TBD (0.25)	0.25	0.50	CH_2_Cl_2_	18	80:20	92
6	TBD (0.25)	0.25	0.50	DMSO	---	---	---
7	TBD (0.25)	0.25	0.50	DMF	---	---	---
8	Cs_2_CO_3_ (0.25)	0.25	0.50	CH_2_Cl_2_	10	86:14	89
9	Et_3_N (1.0)	1.0	1.9	CH_2_Cl_2_	23	83:17	94
10^[e]^	Et_3_N (1.0)	1.0	1.9	CH_2_Cl_2_	56	82:18	95
11^[e]^	Et_3_N (0.25)	0.25	0.50	CH_2_Cl_2_	25	80:20	94
12^[e]^	Et_3_N (1.5)	1.5	3.0	CH_2_Cl_2_	27	79:21	91
13^[e]^	Et_3_N (1.0)	1.0	1.9	CHCl_3_	58	83:17	92
14^[e]^	Et_3_N (1.0)	1.0	1.9	Toluene	38	81:19	92
15^[e]^	Et_3_N (1.0)	1.0	1.9	THF	43	76:24	96

^[a]^ Reactions were performed using **1** (0.20 mmol). ^[b]^ Combined isolated yield of **6a** and *epi*-**6a**. ^[c]^ Ratio of **6a** and *epi*-**6a** determined by ^1^H NMR analysis. ^[d]^ Determined by chiral HPLC analysis. ^[e]^ Conducted at 0 °C.

**Table 2:** Scope of the nucleophile^[a]^.

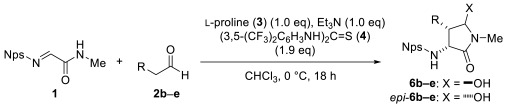				
Entry	R (2)	Yield	dr^[c]^	ee of 6
		(%)^[b]^		(%)^[d]^
1^[e]^	Bu (**2a**)	58	83:17	92
2	*i*-Pr (**2b**)	22	53:47	97
3	PhCH_2_ (**2c**)	21	82:18	89
4	CH_3_(CH_2_)_4_CH=CHCH_2_ (**2d**)	22	81:19	81
5	BocNH(CH_2_)_4_ (**2e**)	28	76:24	89

^[a]^ Reactions were performed using **1** (0.20 mmol). ^[b]^ Combined isolated yield of **6** and *epi*-**6**. ^[c]^ Ratio of **6** and *epi*-**6** determined by ^1^H NMR analysis. ^[d]^ Determined by chiral HPLC analysis. ^[e]^ Data from Table 1, entry 13 for comparison.

**Table 3:** Observed and calculated ^13^C NMR chemical shifts of **6a** and *epi*-**6a** and DP4 analysis.

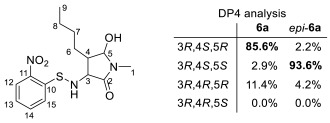						
	Observed (ppm)		Calculated (ppm)			
	6a	*epi*-6a	3*R*,4*S*,5*R*	3*R*,4*S*,5*S*	3*R*,4*R*,5*R*	3*R*,4*R*,5*S*
C-1	27.7	27.7	26.9	26.2	27.2	26.2
C-2	173.1	173.4	174.0	172.1	175.5	173.3
C-3	60.9	63.2	59.2	61.4	61.5	57.8
C-4	45.8	42.9	47.8	47.2	47.0	44.0
C-5	87.1	85.6	88.5	85.1	84.7	91.0
C-6	26.3	23.9	25.6	25.3	25.7	29.3
C-7	29.2	29.6	28.9	28.7	28.3	27.4
C-8	22.8	22.9	22.5	22.5	21.5	20.4
C-9	14.0	14.0	13.9	13.6	13.4	14.6
C-10	144.6	144.5	144.8	143.1	147.1	140.6
C-11	142.8	142.4	144.9	144.1	144.6	147.2
C-12	126.0	125.6	127.4	126.6	127.4	128.2
C-13	124.3	125.1	124.0	125.2	123.9	124.8
C-14	134.2	134.1	133.1	133.3	133.1	132.8
C-15	125.0	125.0	126.0	128.2	125.7	126.7

## Data Availability

The original contributions presented in this study are included in the Supplementary Materials. Further inquiries can be directed to the corresponding authors.

## Supplementary Materials

The following supporting information can be downloaded at https://www.mdpi.com/article/10.3390/molecules31030449/s1, The copies of ^1^H- and ^13^C-NMR spectra, and chiral HPLC charts are available online.
