# Supplementary material for: Asymmetric Mannich Reaction of α-(2-Nitrophenylsulfenyl)imino Acetamide: A Cyclization-Driven Process
Source: Molecules. 2026 Jan 27;31(3):449. doi: 10.3390/molecules31030449 (PMC12899788; doi:10.3390/molecules31030449)
Supplement: Supplementary file 1 [file molecules-31-00449-s001.zip › molecules-4048175-supplementary.pdf]

Supplementary Materials

Asymmetric Mannich Reaction of α-(2-Nitrophenylsulfenyl)imino Acetamide: A Cyclization-Driven Process

Tsubasa Inokuma [1,2,3,*], Maki Miyamoto [1], Kazuki Okada [1], Genki Nagai [1] and Ken-ichi Yamada [1,2,*]

[1] Graduate School of Pharmaceutical Sciences, Tokushima University, Shomachi, Tokushima 770-8505, Japan
[2] Research Cluster on "Key Material Development", Tokushima University, Shomachi, Tokushima 770-8505, Japan
[3] Research Cluster on "Hybrid Modality Exploration", Tokushima University, Shomachi, Tokushima 770-8505, Japan
* Correspondence: tinokuma@tokushima-u.ac.jp (T.I.); yamak@tokushima-u.ac.jp (K.Y.)

Contents

1. Copies of ^{1}H and ^{13}C NMR spectra.. S2–14

2. Copies of NOESY NMR spectra of **6a** and *epi*-**6a** S15

3. Copies of HPLC charts S16–21

Supplementary Materials

1. Copies of ^{1}H and ^{13}C NMR spectra

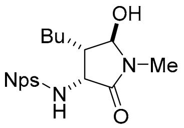

6a

^{1}H NMR spectrum of **6a** (500 MHz in $CDCl_3$)

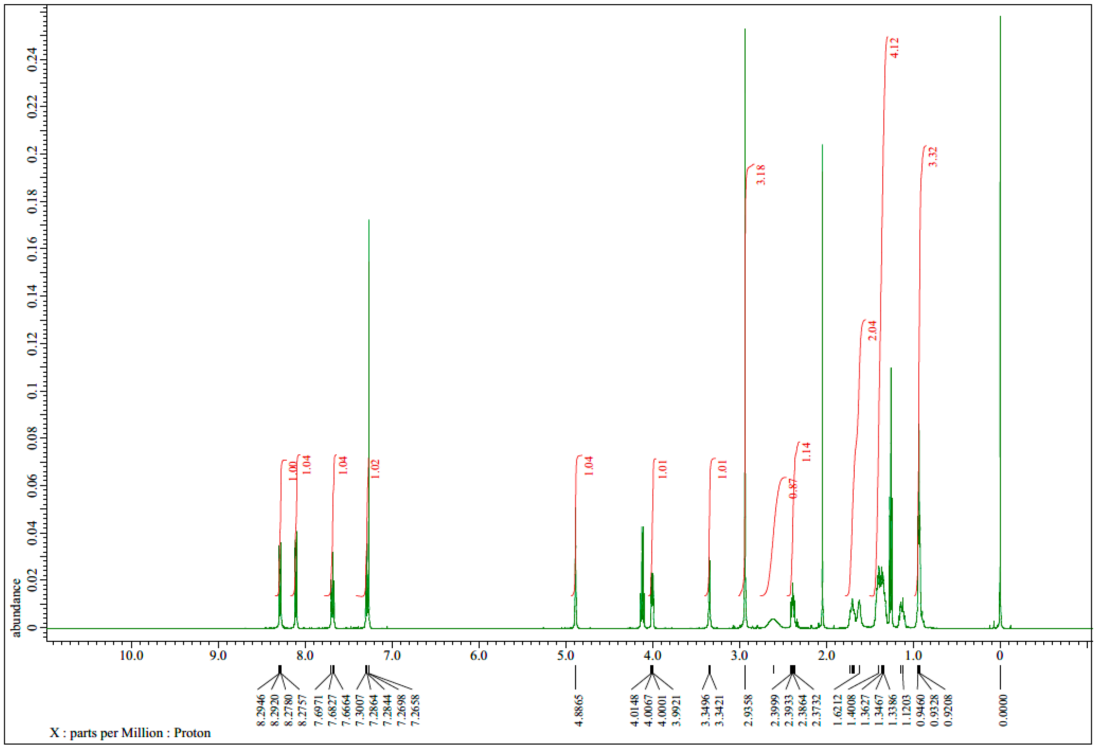

^{13}C NMR spectrum of **6a** (125 MHz in $CDCl_3$)

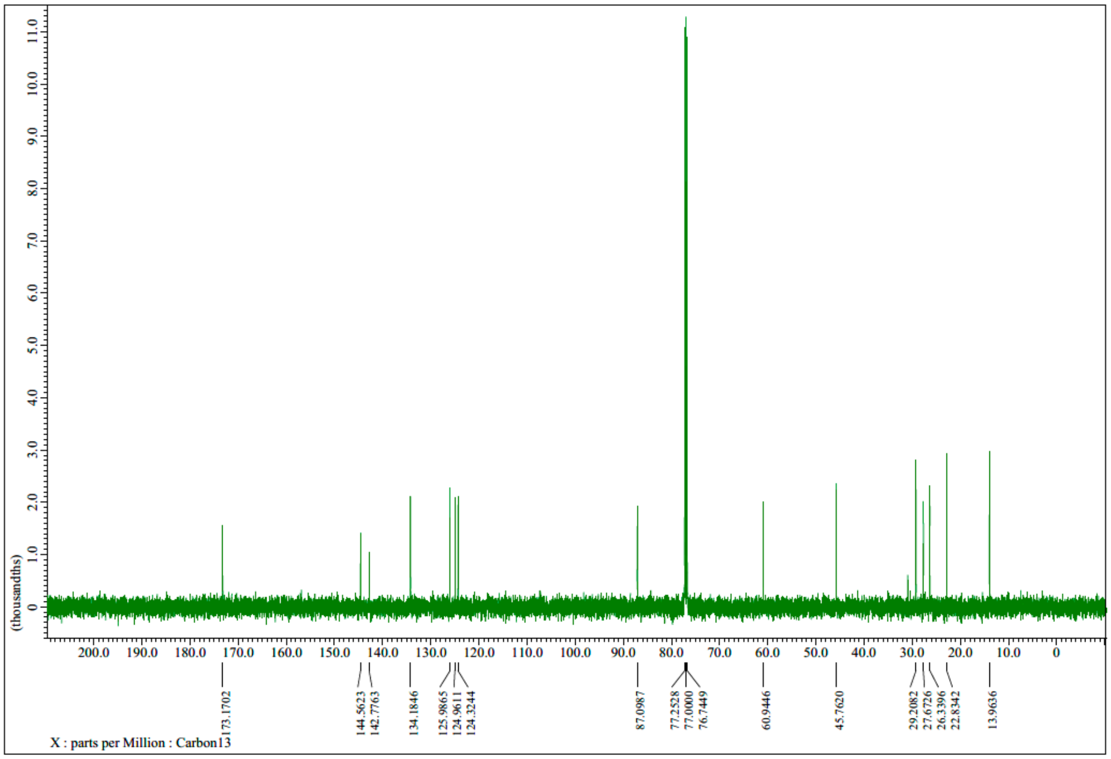

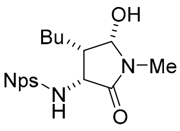

epi-**6a**

^{1}H NMR spectrum of *epi*-**6a** (500 MHz in $CDCl_3$)

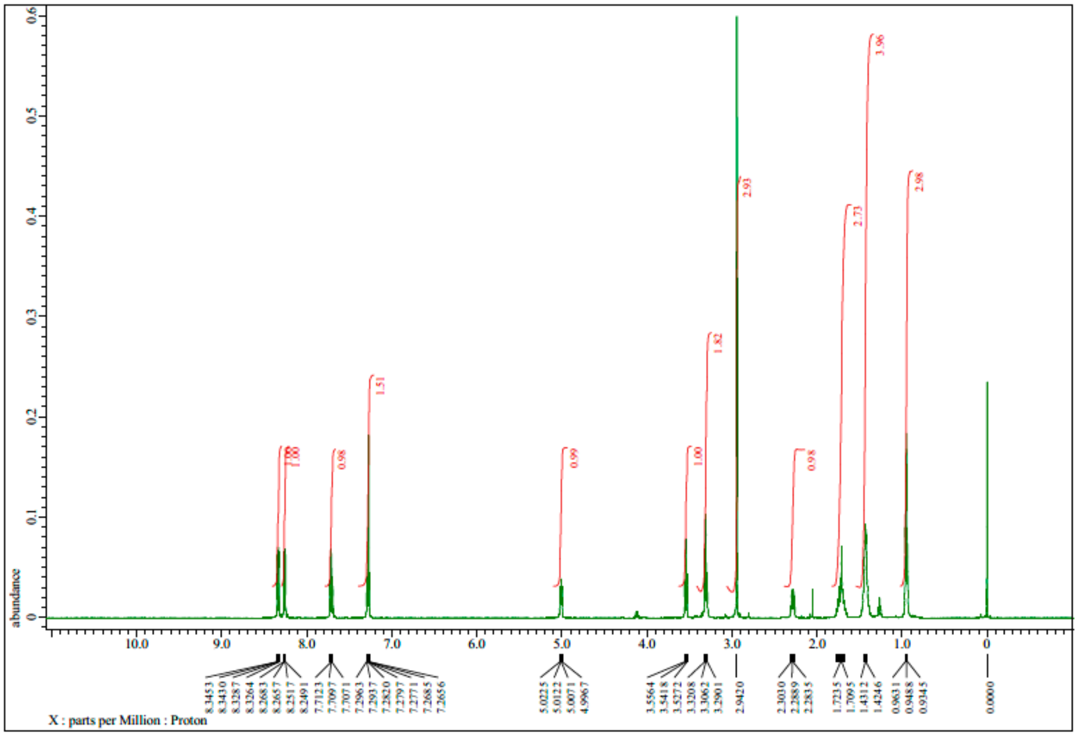

^{13}C NMR spectrum of *epi*-**6a** (125 MHz in $CDCl_3$)

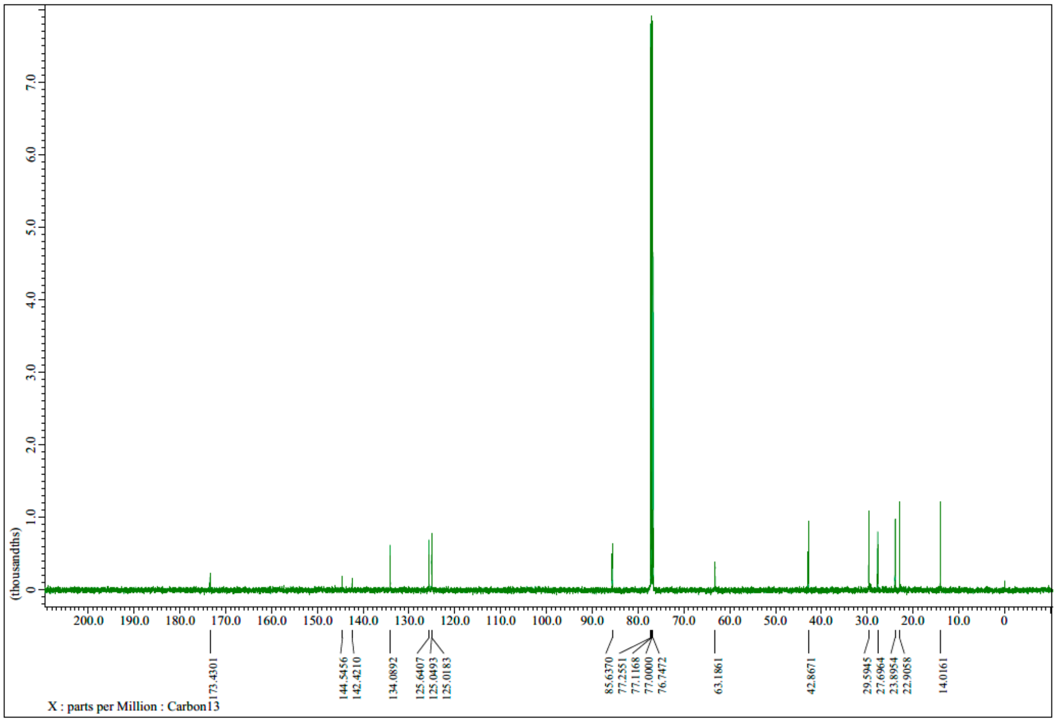

Supplementary Materials

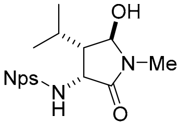

6b

^{1}H NMR spectrum of **6b** (500 MHz in $CDCl_3$)

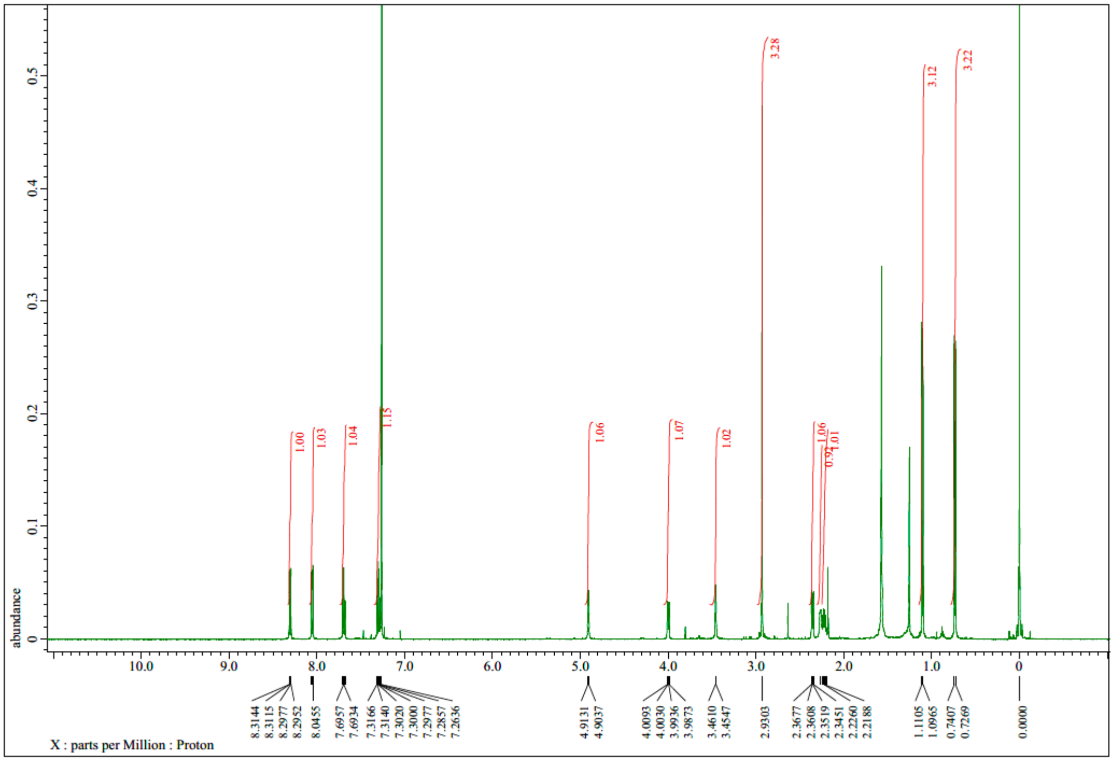

^{13}C NMR spectrum of **6b** (125 MHz in $CDCl_3$)

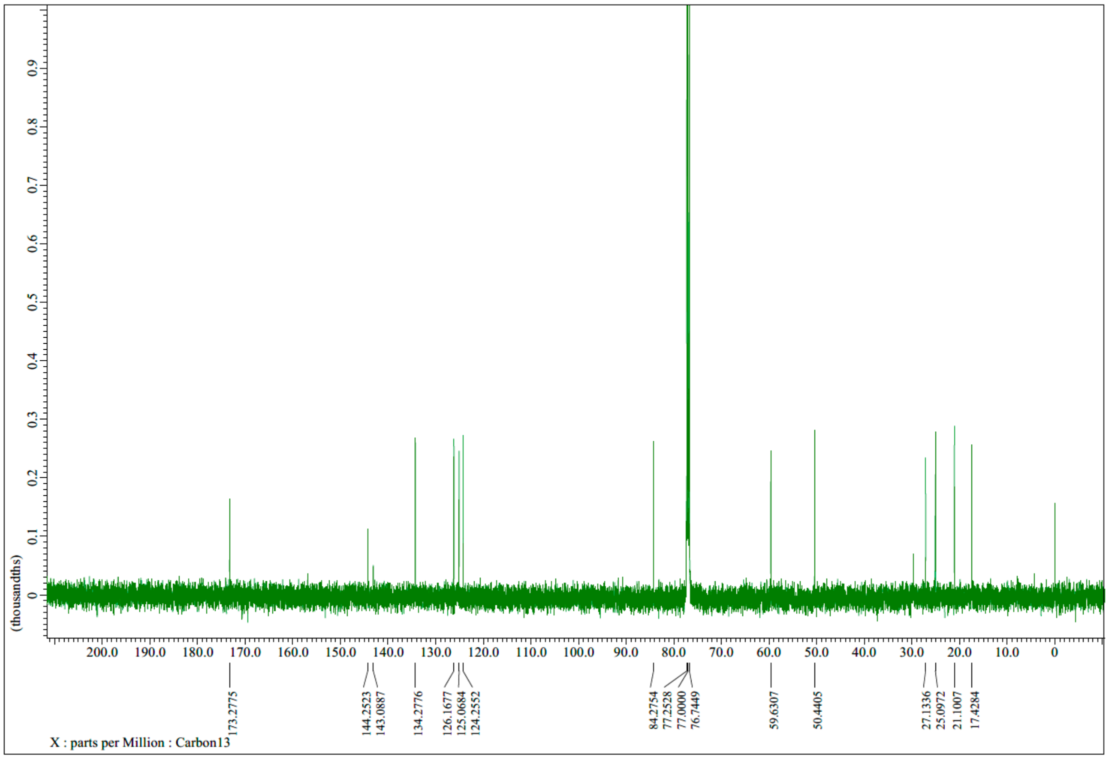

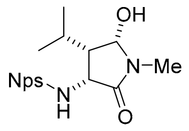

epi-**6b**

^{1}H NMR spectrum of *epi*-**6b** (400 MHz in $CDCl_3$)

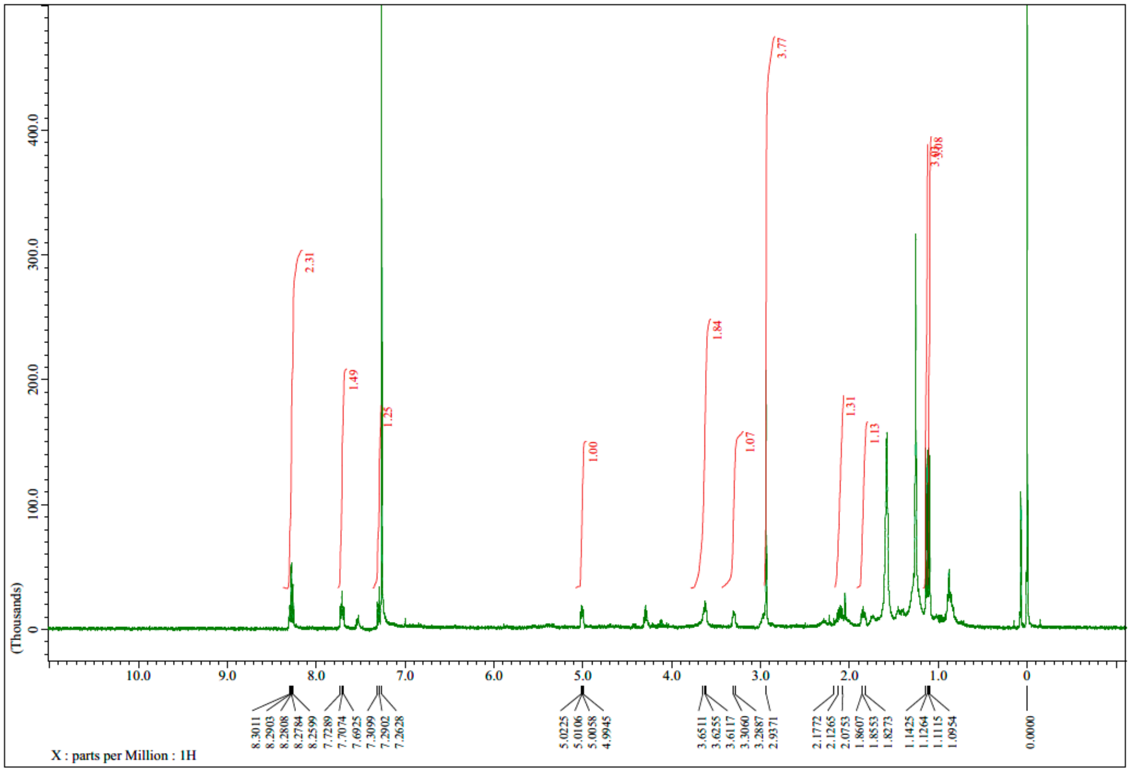

^{13}C NMR spectrum of *epi*-**6b** (125 MHz in $CDCl_3$)

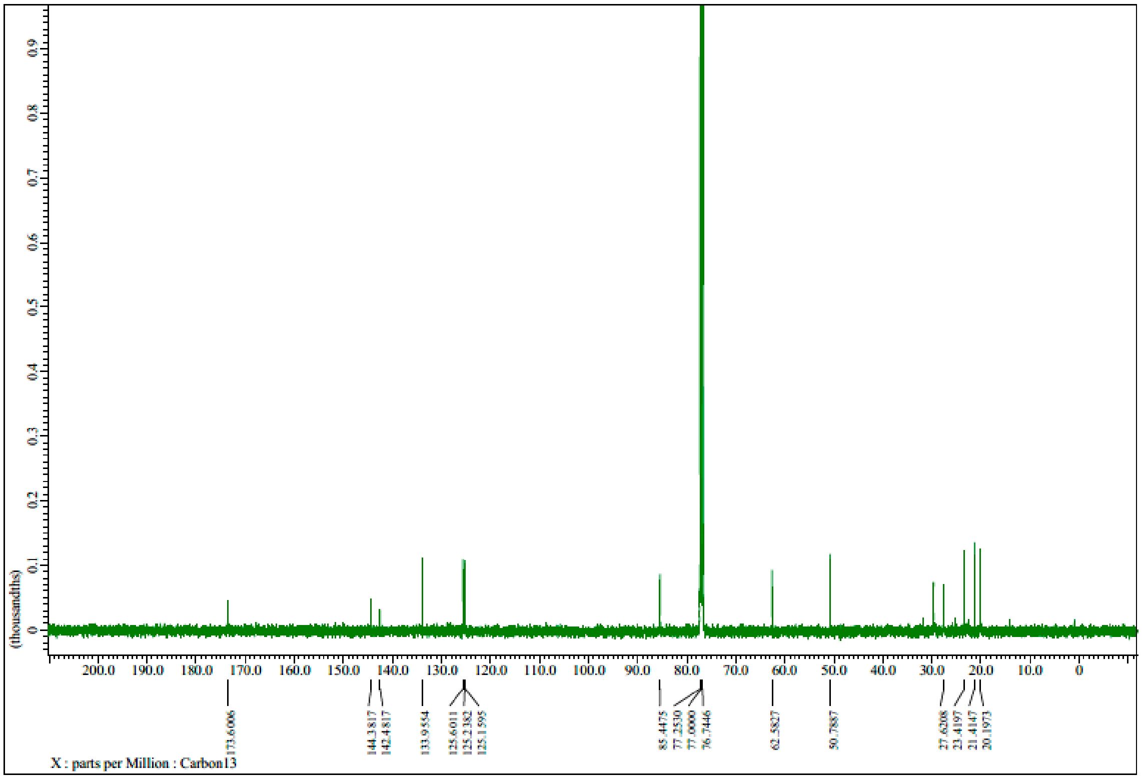

Supplementary Materials

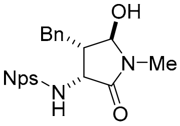

6c

^{1}H NMR spectrum of **6c** (500 MHz in $CDCl_3$)

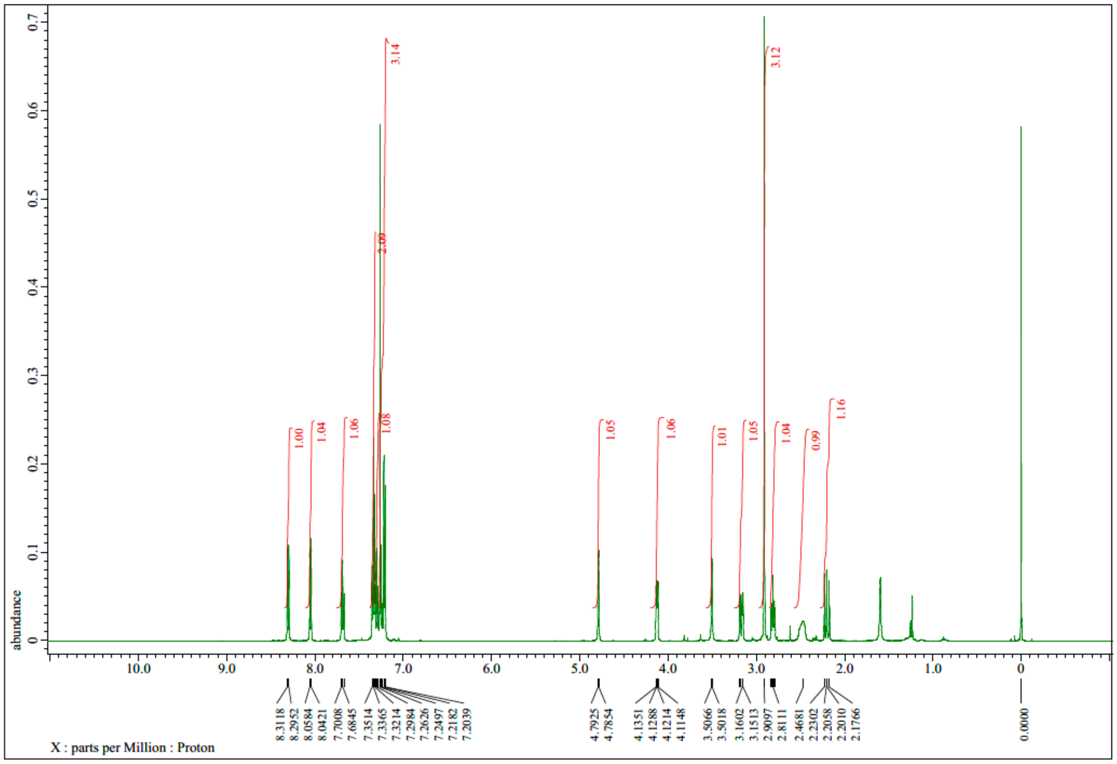

^{13}C NMR spectrum of **6c** (100 MHz in $CDCl_3$)

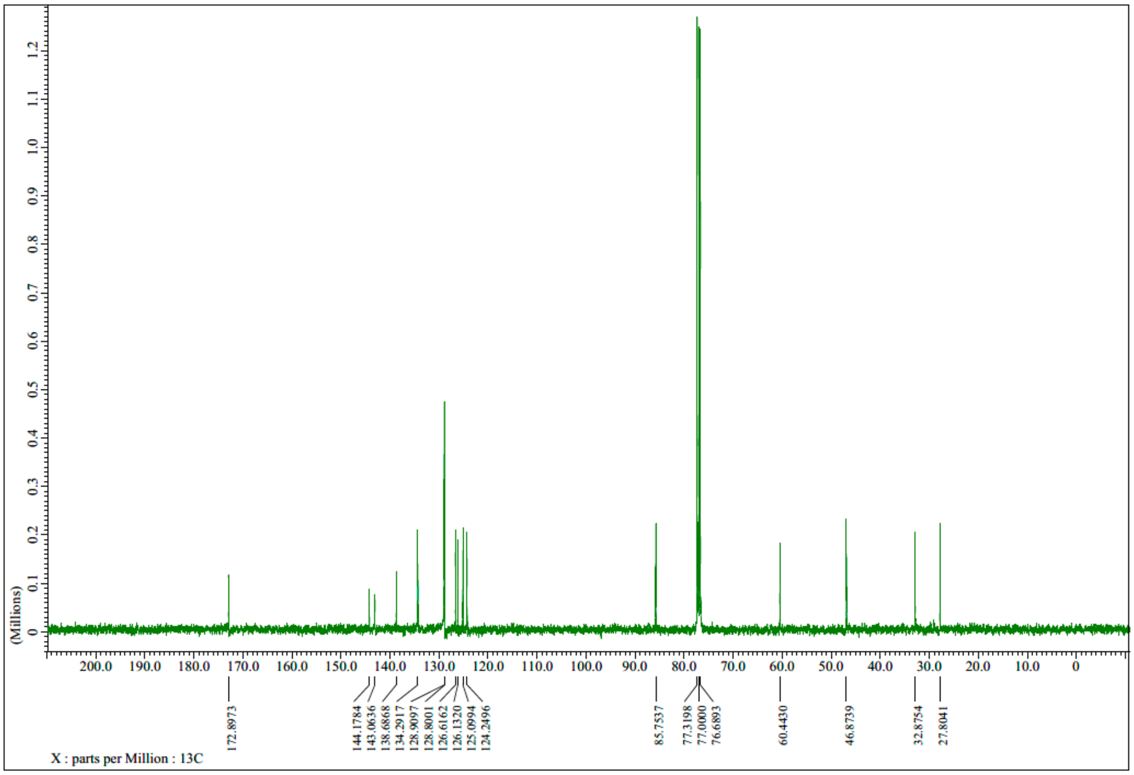

Supplementary Materials

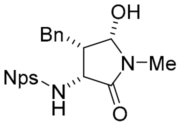

epi-**6c**

^{1}H NMR spectrum of *epi*-**6c** (400 MHz in CDCl$_3$)

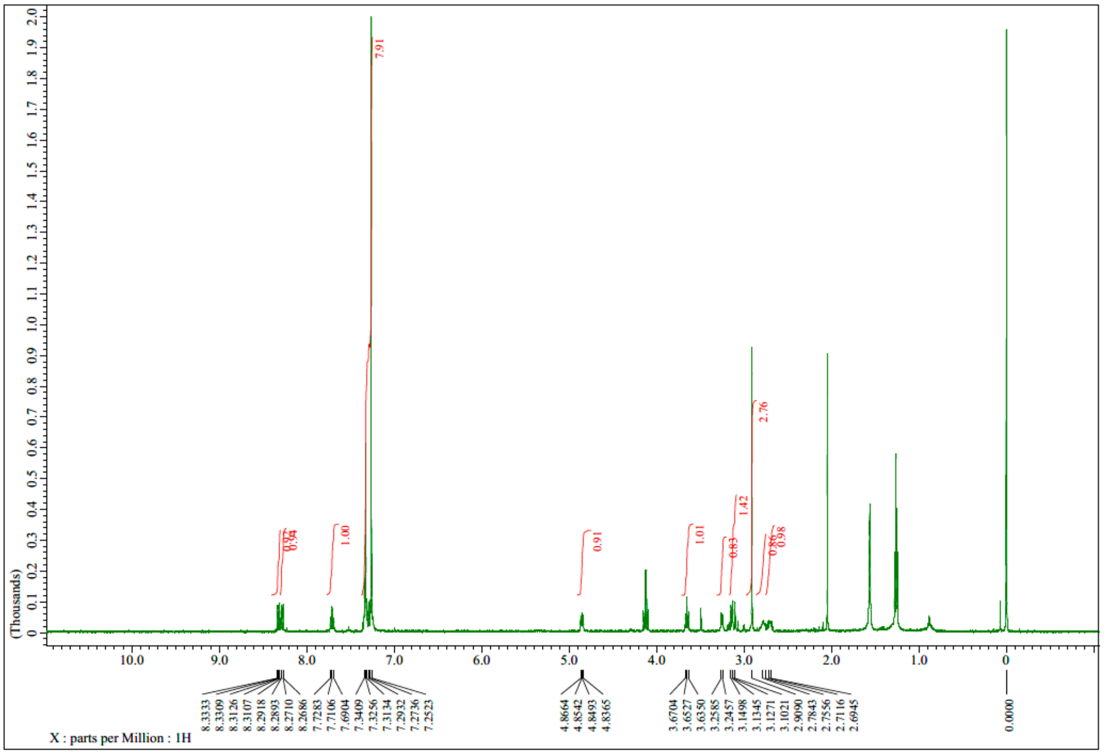

^{13}C NMR spectrum of *epi*-**6c** (100 MHz in CDCl$_3$)

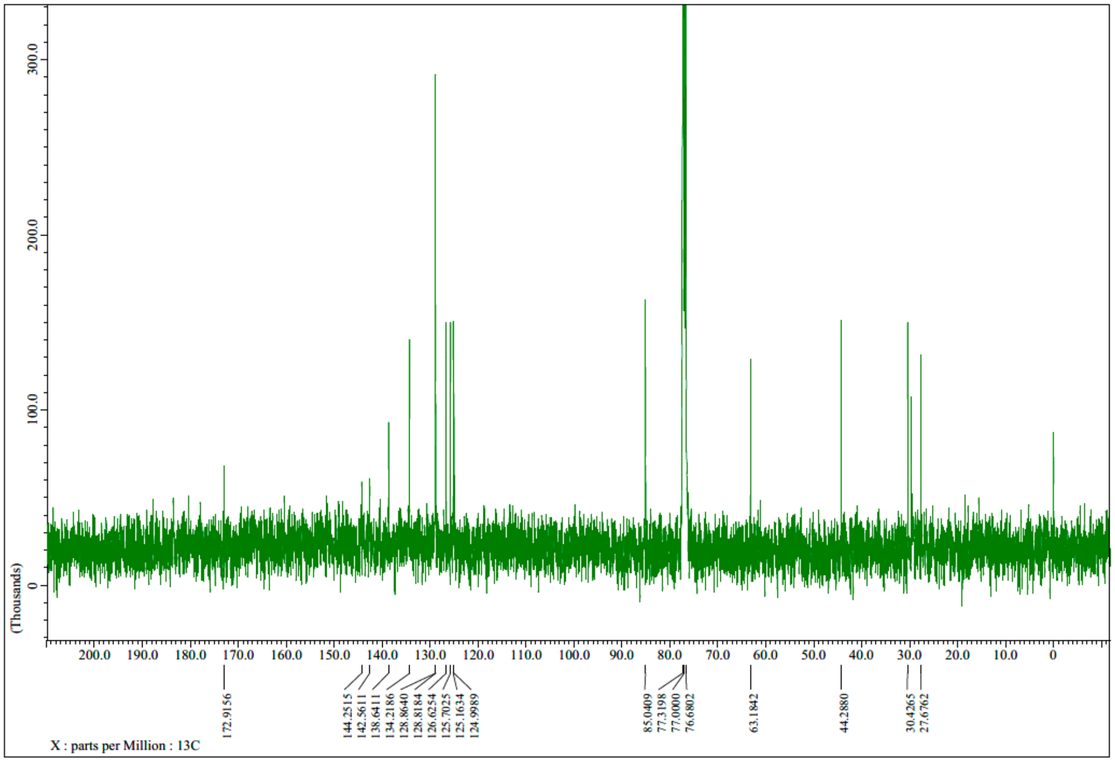

Supplementary Materials

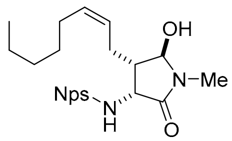

6d

^{1}H NMR spectrum of **6d** (500 MHz in $CDCl_3$)

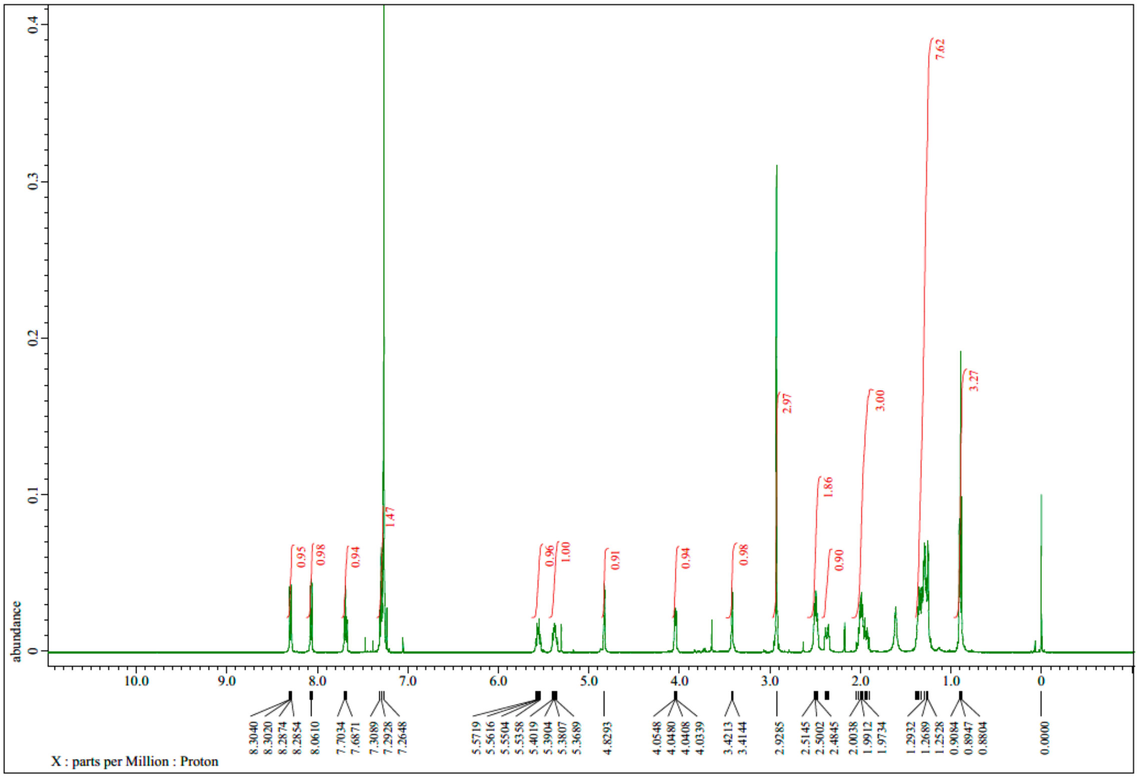

^{13}C NMR spectrum of **6d** (100 MHz in $CDCl_3$)

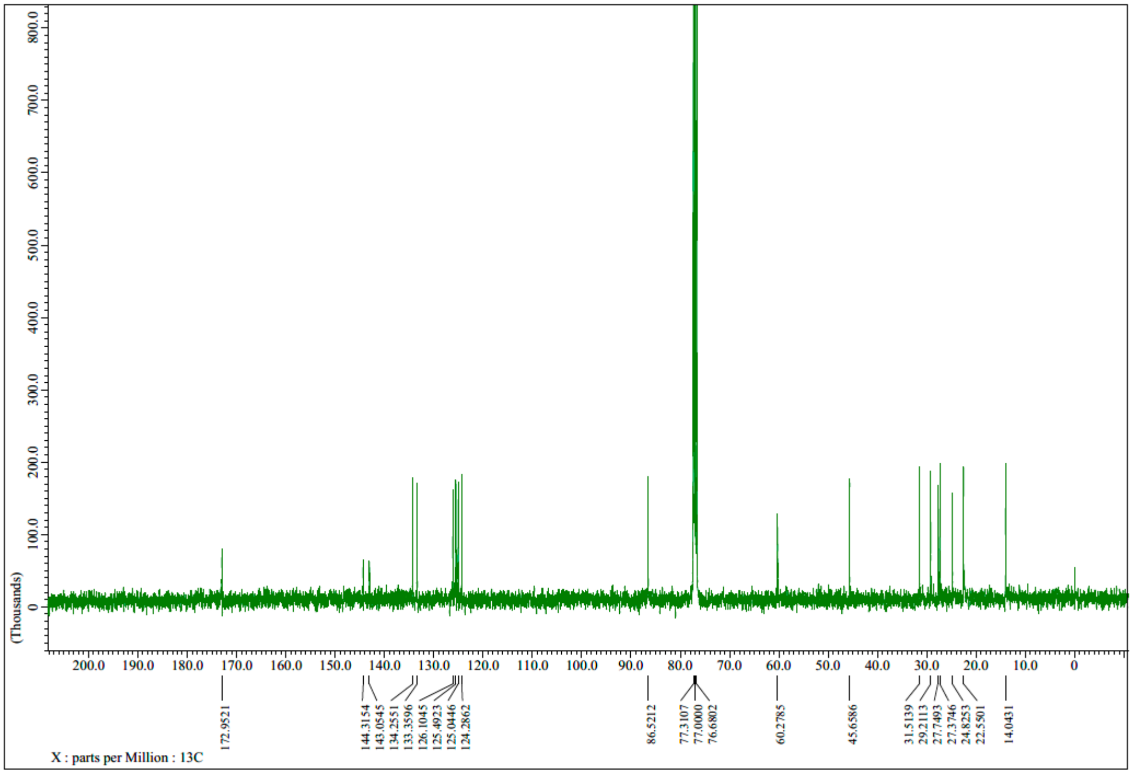

Supplementary Materials

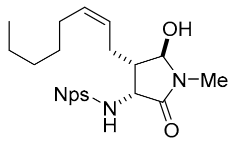

epi-**6d**

^{1}H NMR spectrum of *epi*-**6d** (400 MHz in $CDCl_3$)

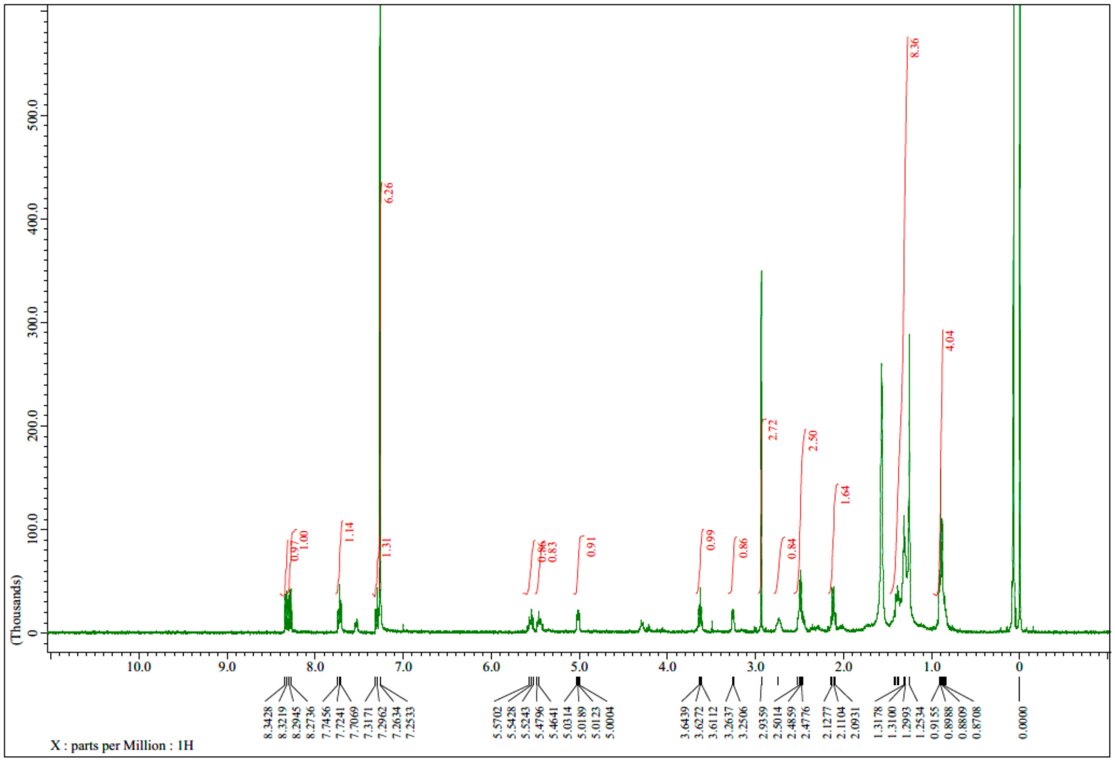

^{13}C NMR spectrum of *epi*-**6d** (125 MHz in $CDCl_3$)

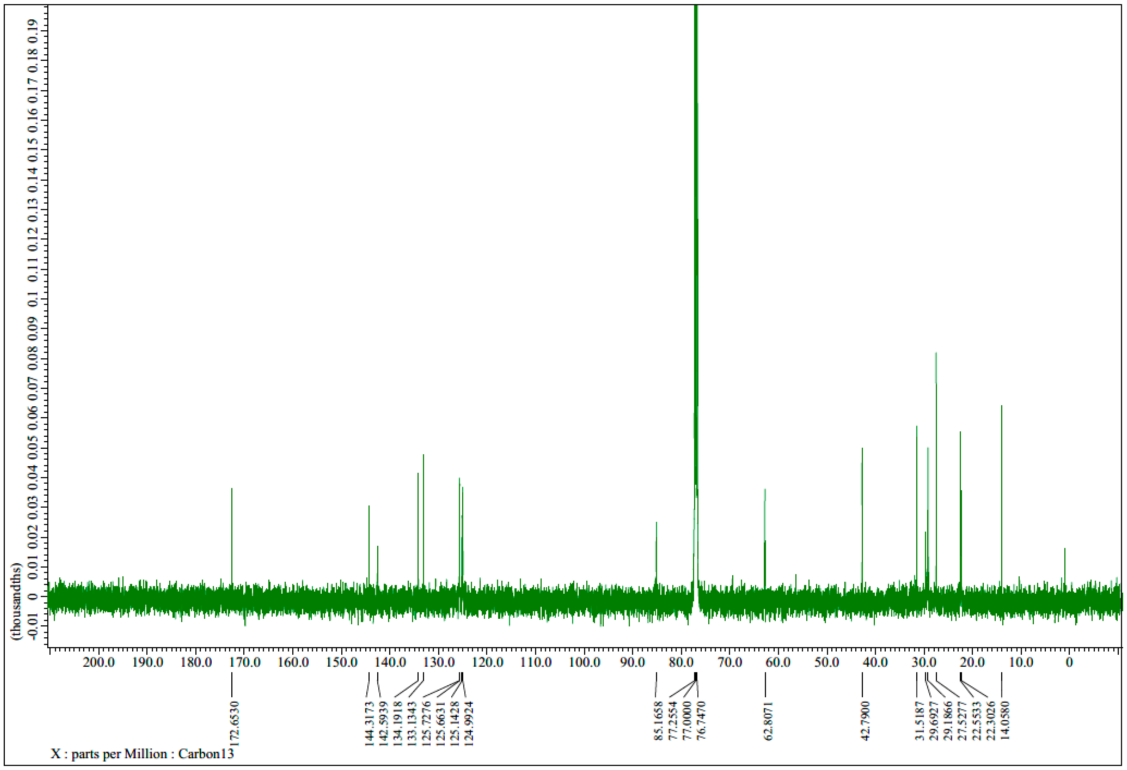

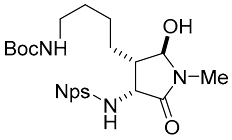

6e

^{1}H NMR spectrum of **6e** (400 MHz in $CDCl_3$)

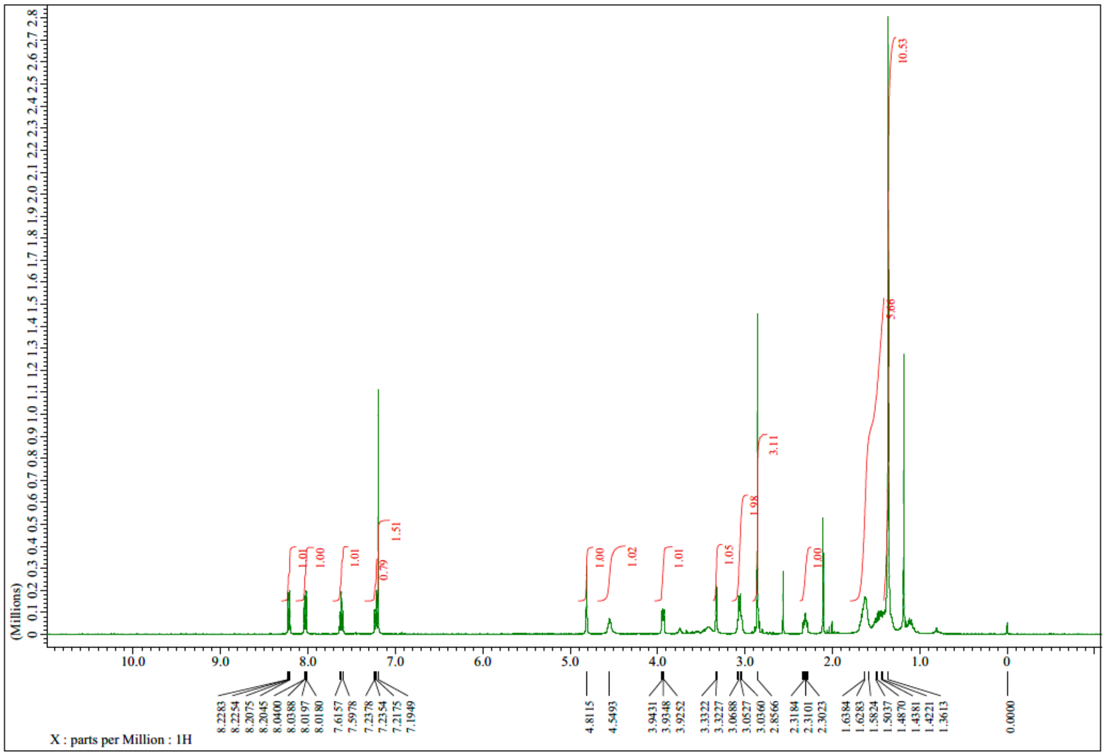

^{13}C NMR spectrum of **6e** (125 MHz in $CDCl_3$)

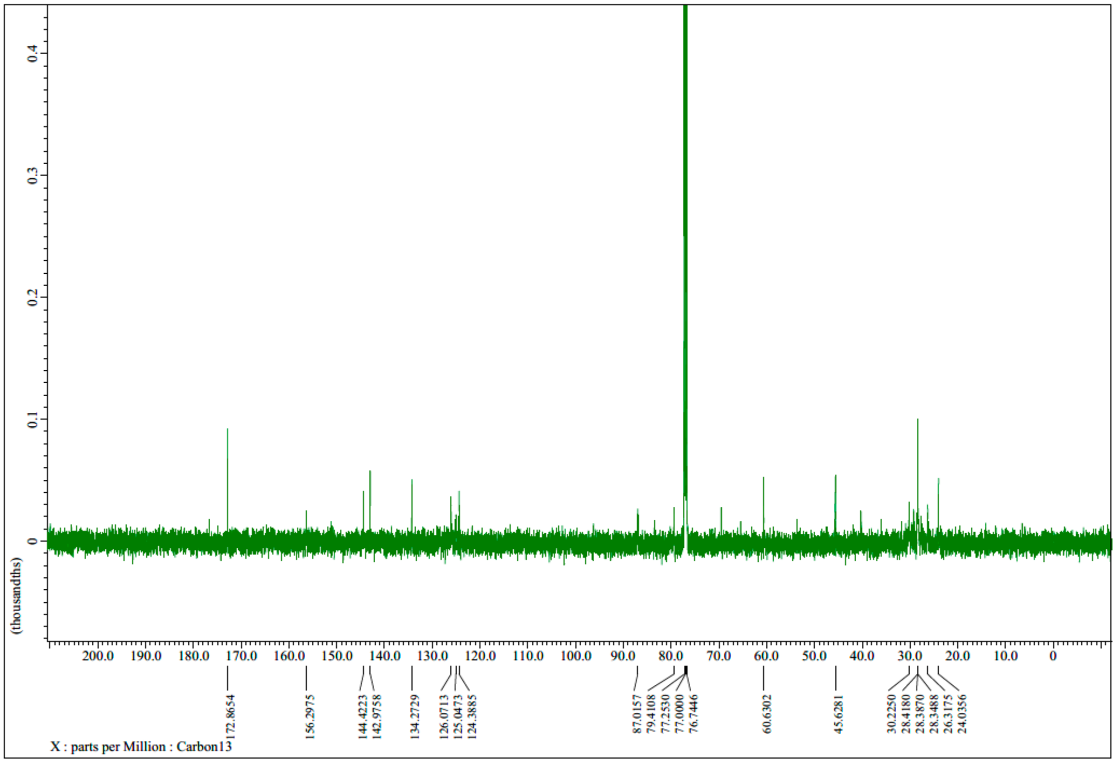

Supplementary Materials

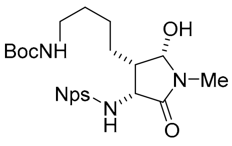

epi-**6e**

^{1}H NMR spectrum of *epi*-**6e** (400 MHz in $CDCl_3$)

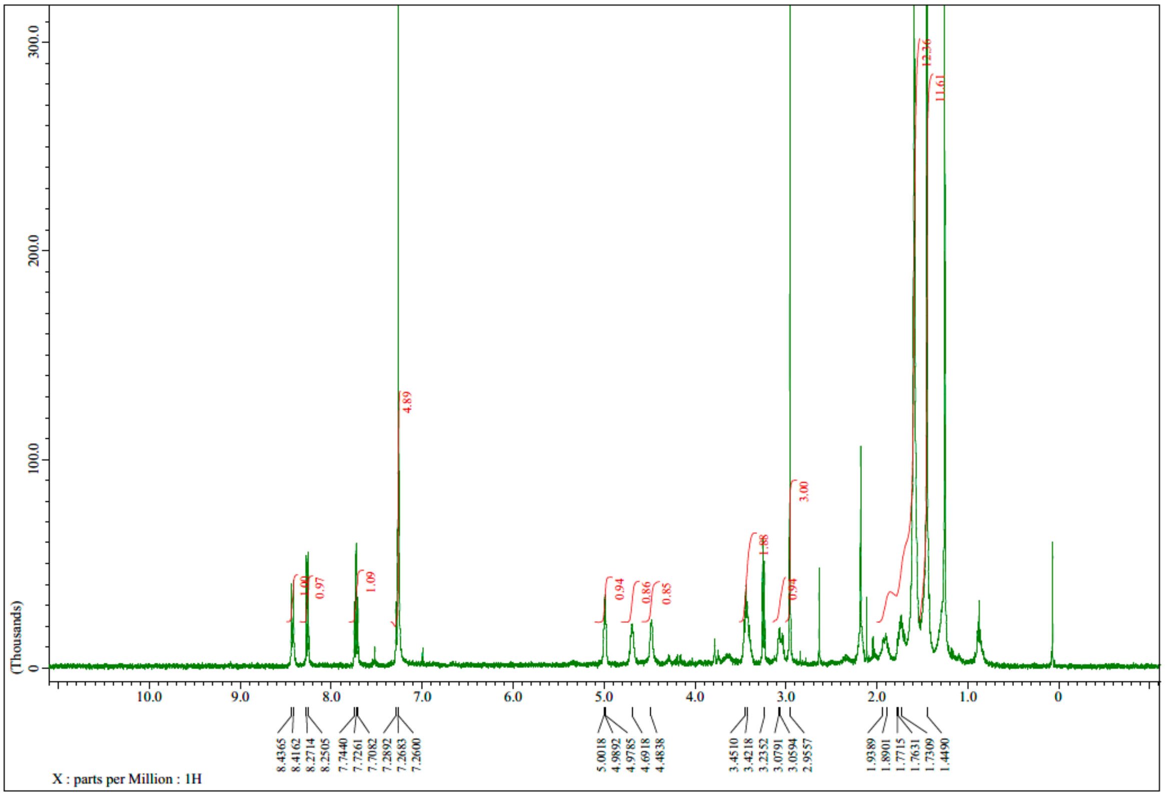

^{13}C NMR spectrum of *epi*-**6e** (125 MHz in $CDCl_3$)

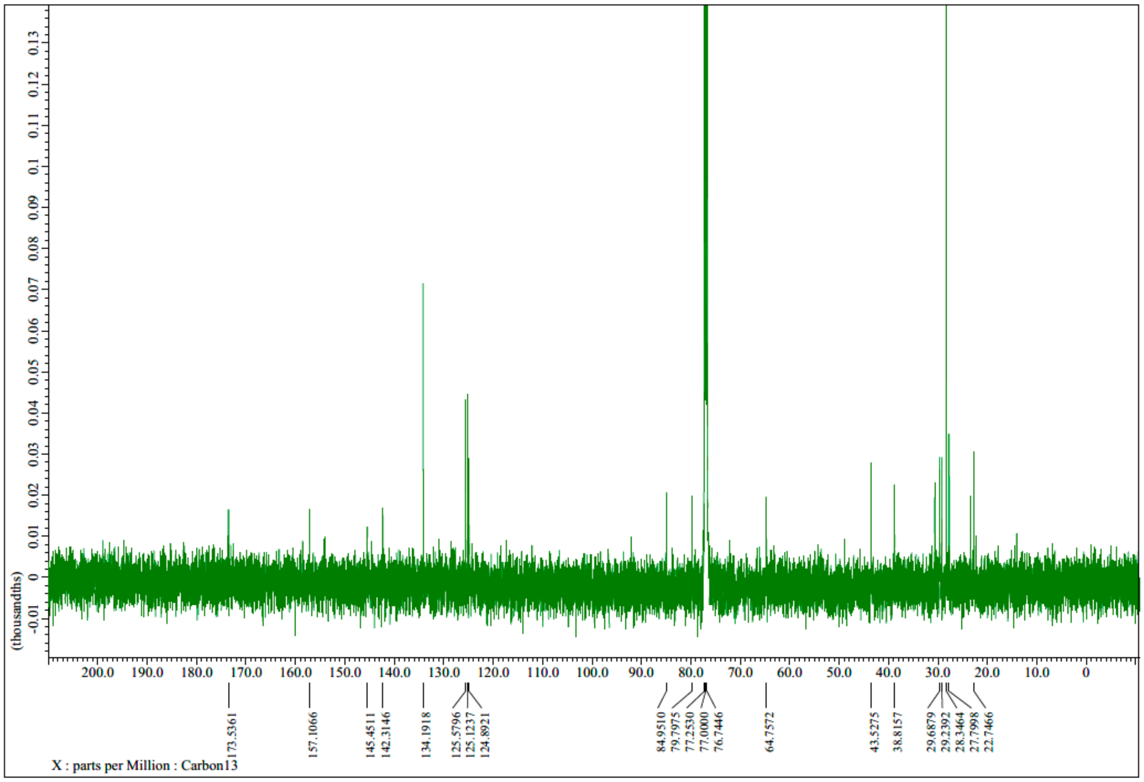

Supplementary Materials

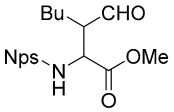

10

^{1}H NMR spectrum of **10** (400 MHz in $CDCl_3$)

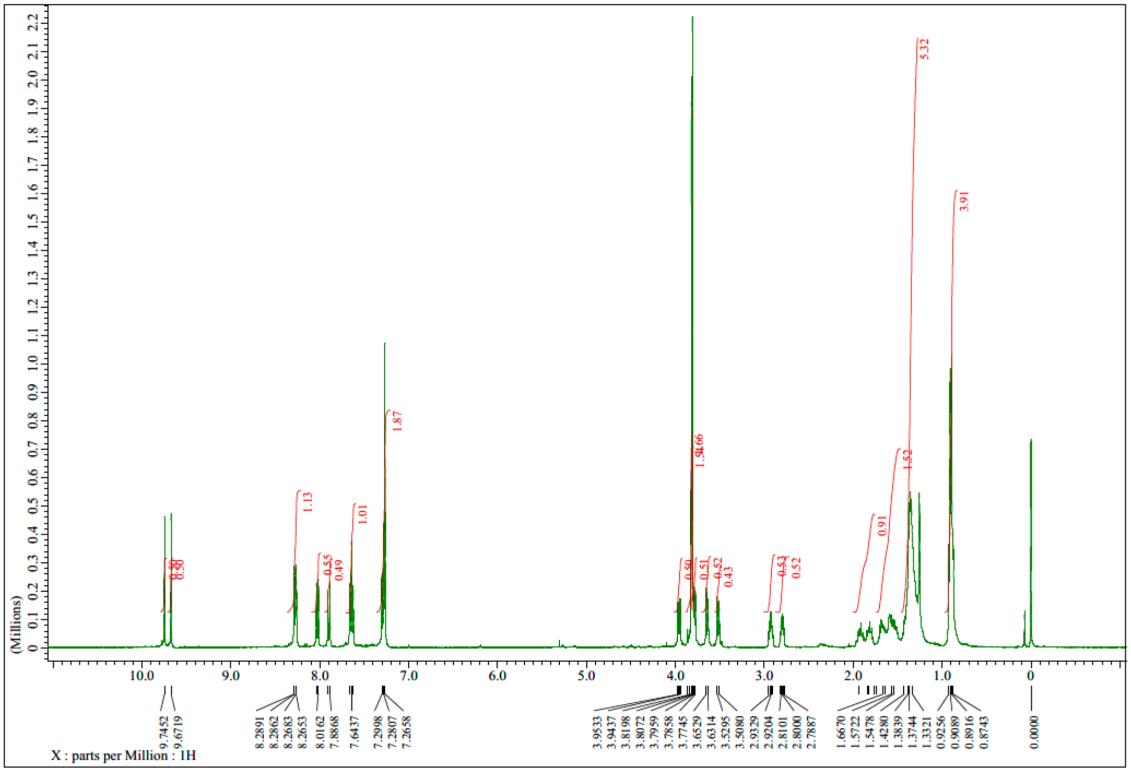

^{13}C NMR spectrum of **10** (100 MHz in $CDCl_3$)

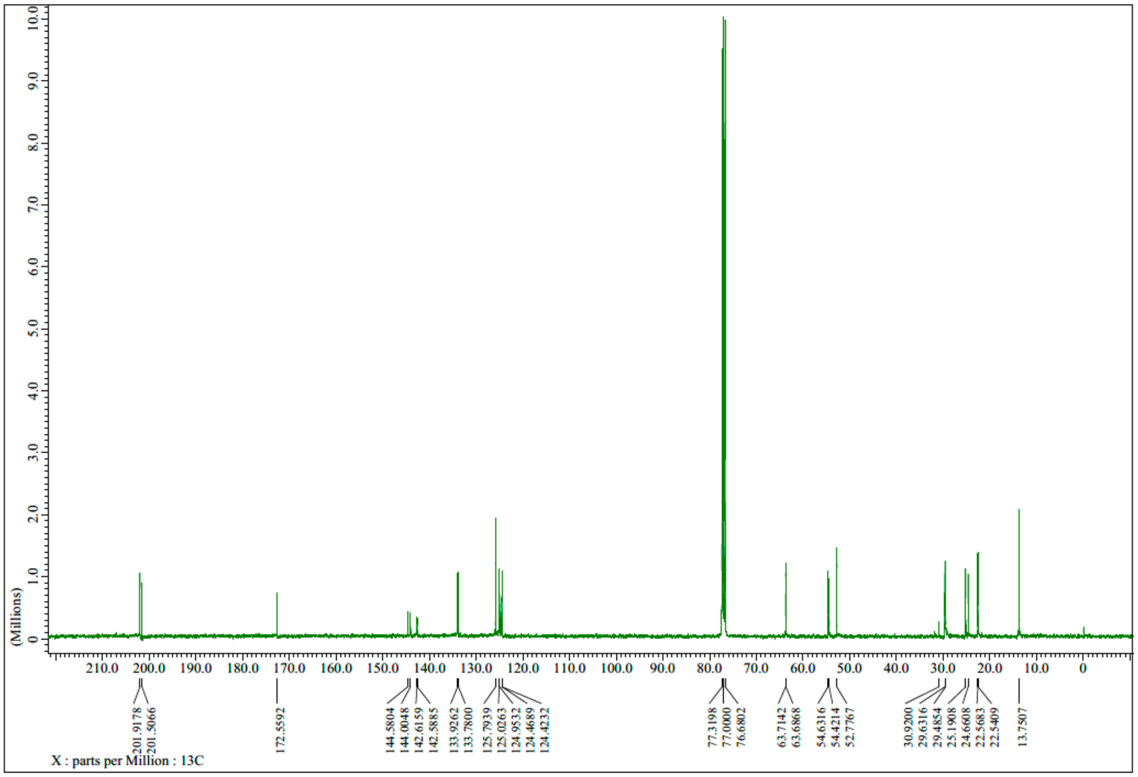

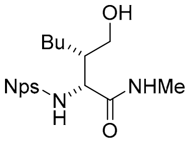

11

^{1}H NMR spectrum of **11** (500 MHz in $CDCl_3$)

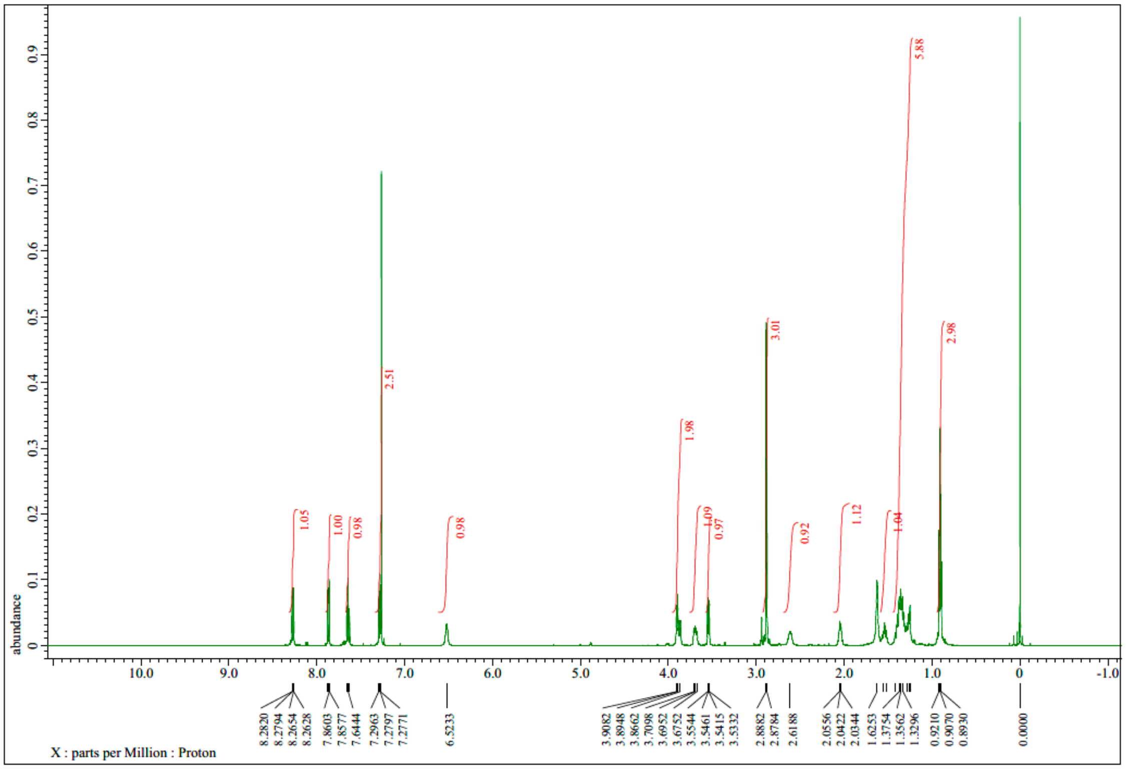

^{13}C NMR spectrum of **11** (125 MHz in $CDCl_3$)

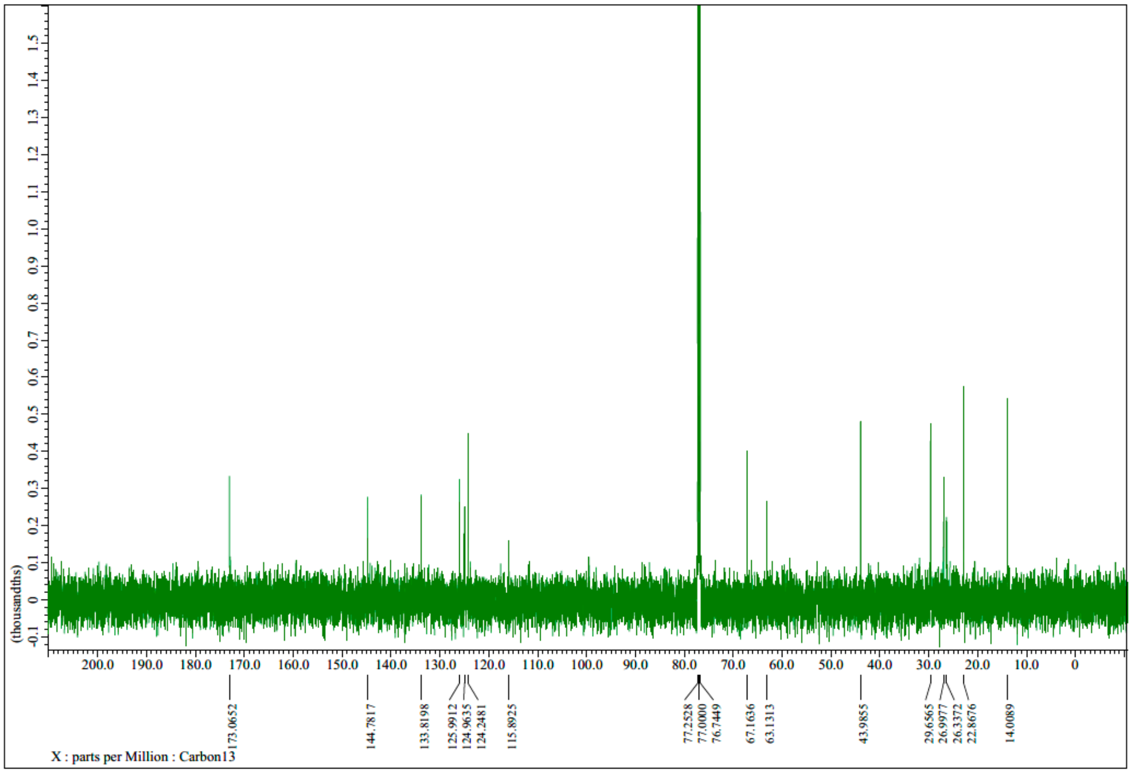

Supplementary Materials

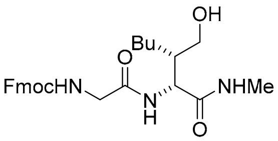

12

^{1}H NMR spectrum of **12** (500 MHz in DMSO-d_6)

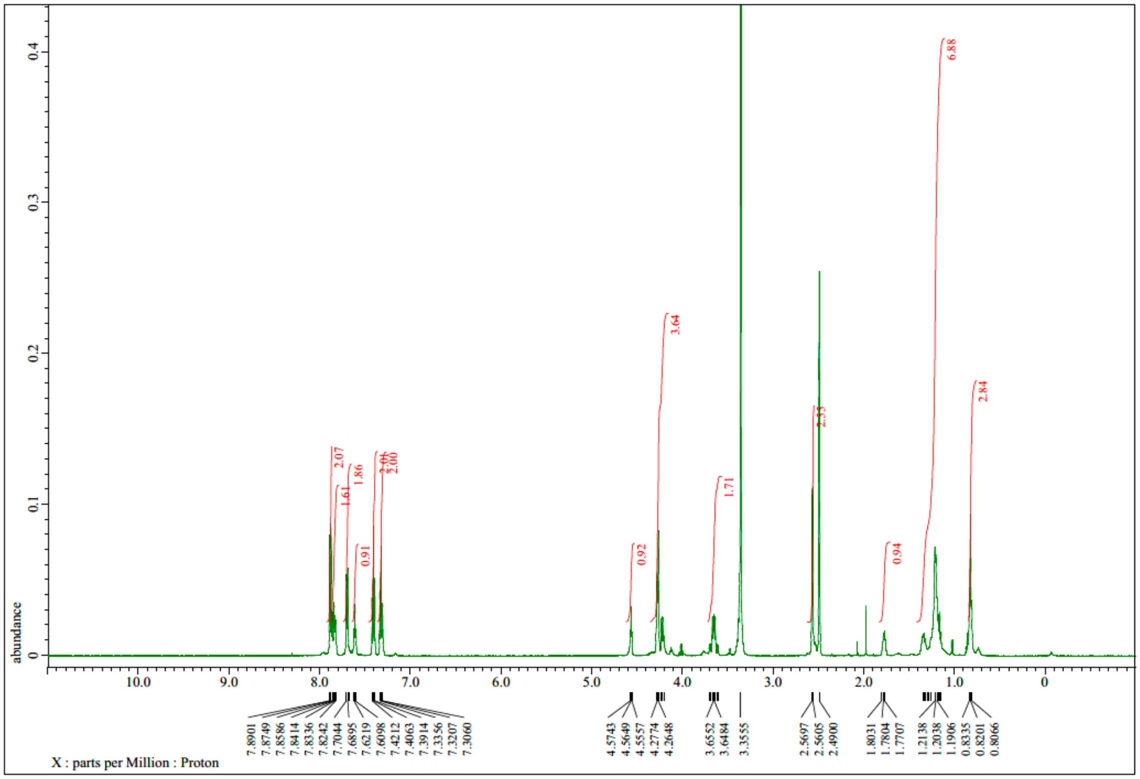

^{13}C NMR spectrum of **12** (125 MHz in DMSO-d_6)

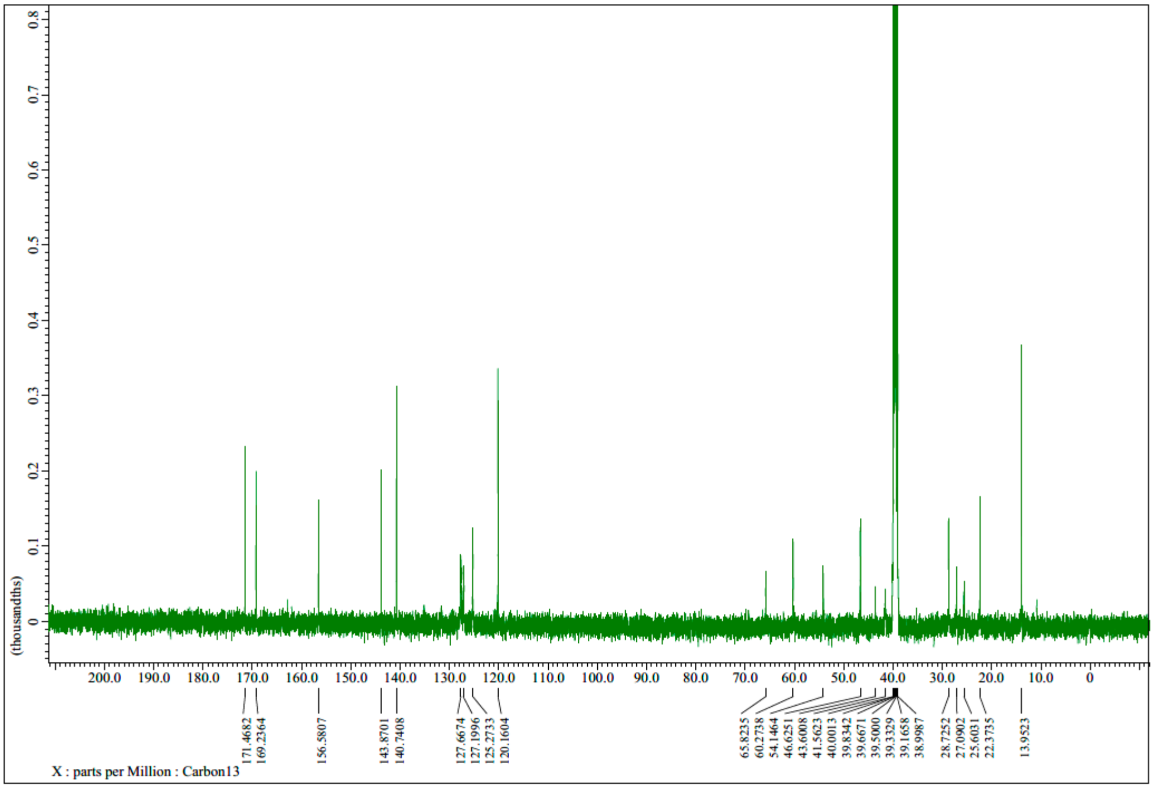

2. Copies of NOESY NMR spectra of **6a** and *epi*-**6a**

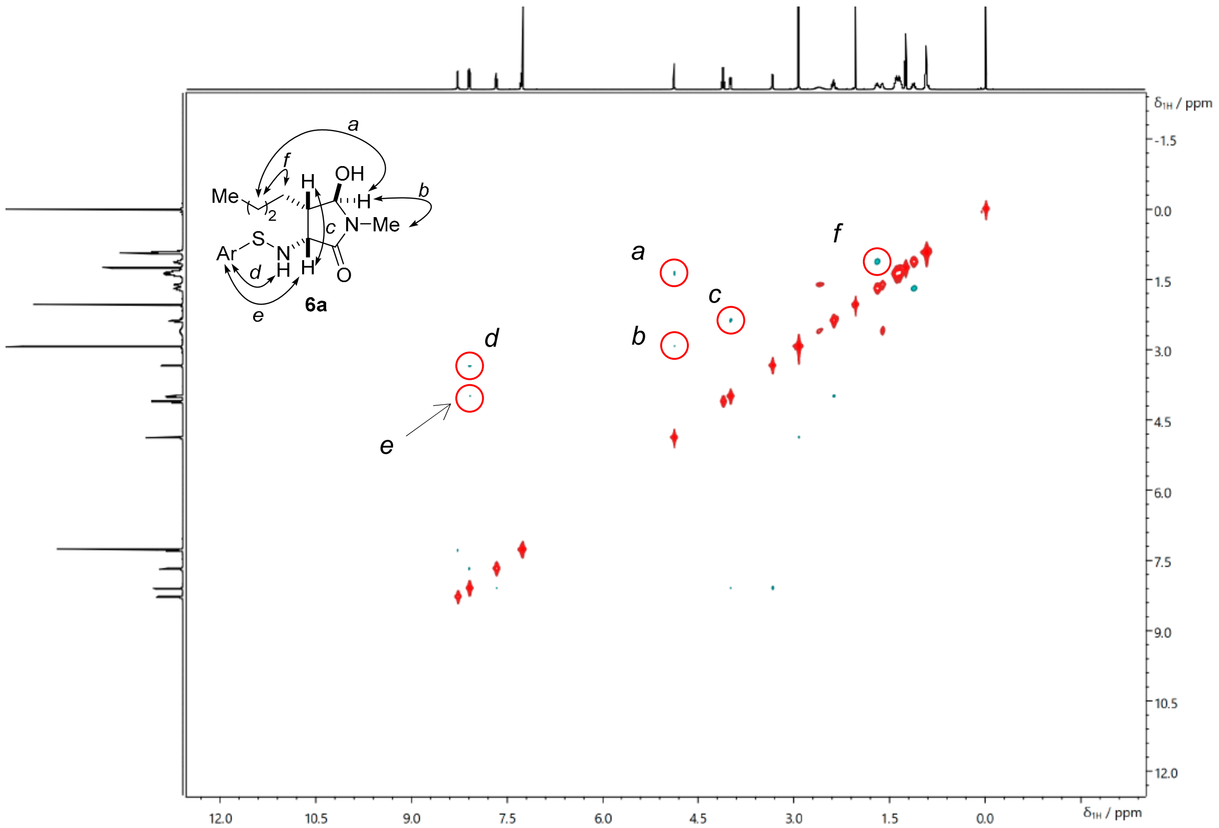

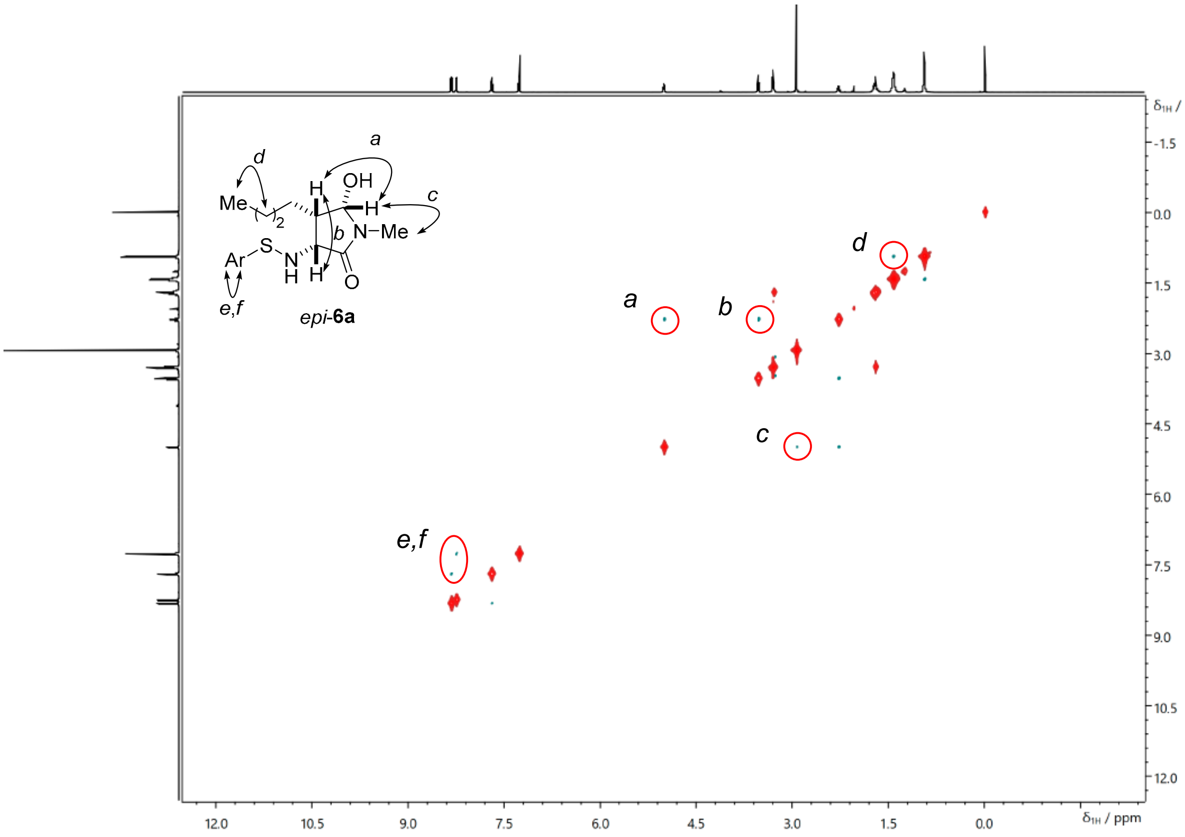

3. Copies of HPLC charts

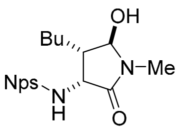

6a

(Daicel chiralpak ADH; hexane/*i*-PrOH 90:10; 1.0 mL/min; 254 nm)

racemate

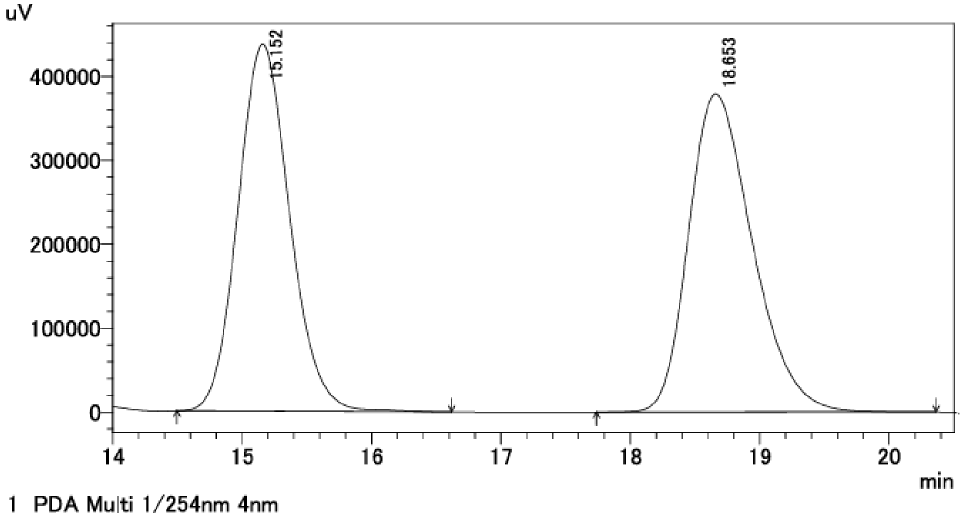

1 PDA Multi 1/254nm 4nm

<Peak Report>

Peak Table

PDA Ch1 254nm 4nm

Peak#	retention time	area	height	area%	height%
1	15.152	12178059	437165	48.370	53.554
2	18.653	12999045	379148	51.630	46.446

6a (92% ee) in Table 1, entry 13

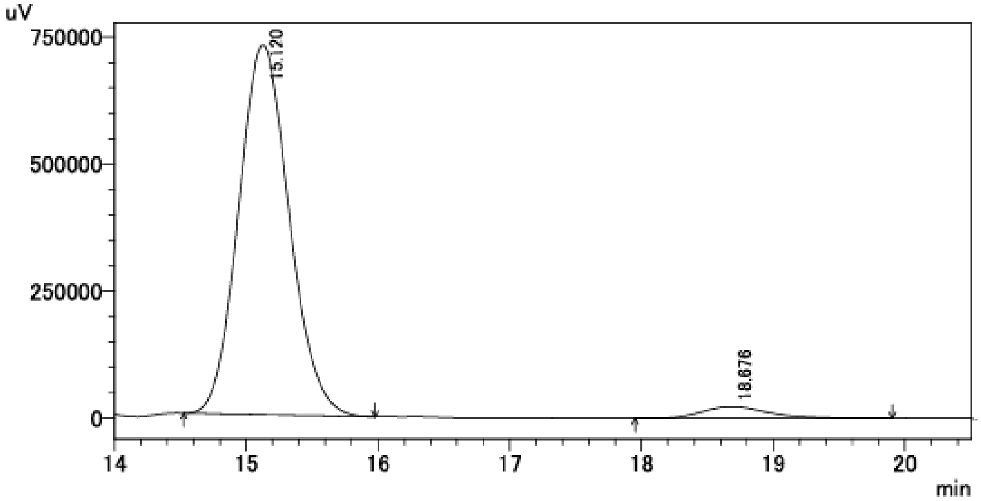

1 PDA Multi 1/254nm 4nm

<Peak Report>

Peak Table

PDA Ch1 254nm 4nm

Peak#	retention time	area	height	area%	height%
1	15.120	19417344	728127	96.183	97.006
2	18.676	770548	22472	3.817	2.994

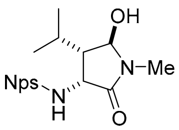

6b

(Daicel chiralpak ADH; hexane/*i*-PrOH 92.5:7.5; 1.0 mL/min; 254 nm)

racemate

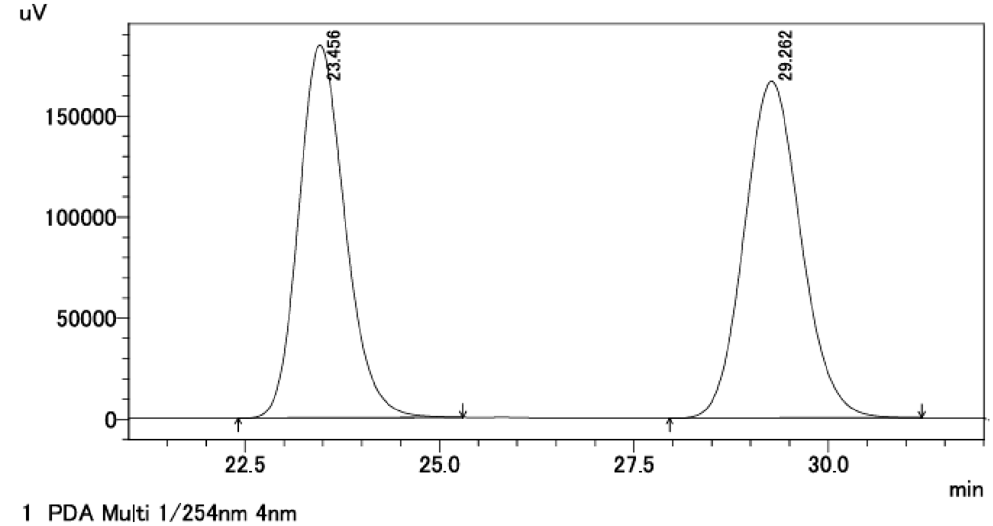

1 PDA Multi 1/254nm 4nm

<Peak Report>

Peak Table

PDA Ch1 254nm 4nm

Peak#	retention time	area	height	area%	height%
1	23.456	7664429	184505	47.574	52.536
2	29.262	8446115	166693	52.426	47.464
[illegible]		[illegible]	[illegible]	[illegible]	[illegible]

6b (97% ee) in Table 2, entry 2

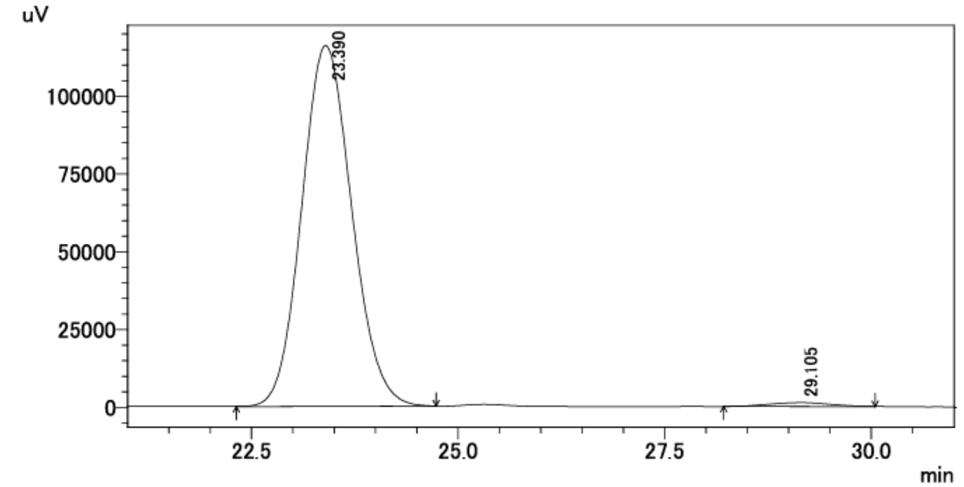

1 PDA Multi 1/254nm 4nm

<Peak Report>

Peak Table

PDA Ch1 254nm 4nm

Peak#	retention time	area	height	area%	height%
1	23.390	4890201	115978	98.656	98.907
2	29.105	66637	1281	1.344	1.093
[illegible]					

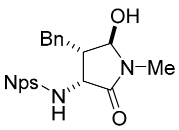

6c

(Daicel chiralpak ADH; hexane/*i*-PrOH 90:10; 1.0 mL/min; 254 nm)

racemate

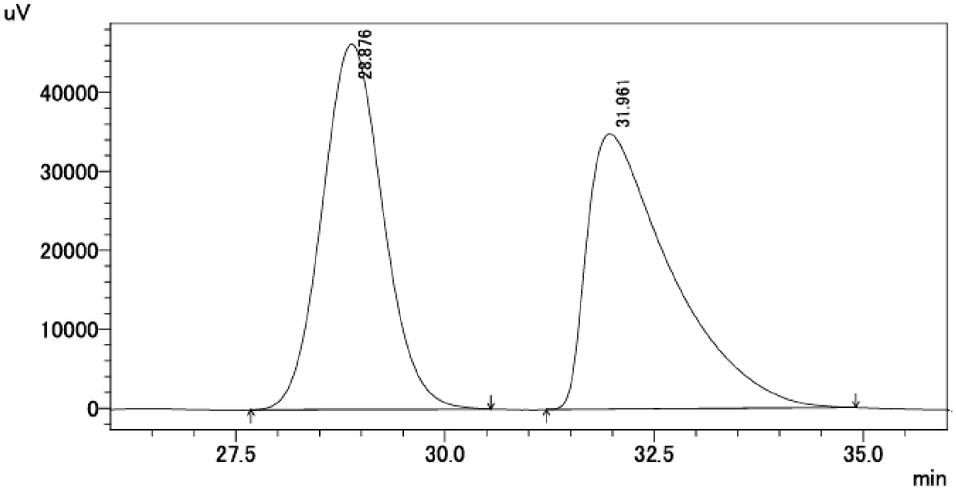

1 PDA Multi 1/254nm 4nm

<Peak Report>

Peak Table

PDA Ch1 254nm 4nm

Peak#	retention time	area	height	area%	height%
1	28.876	2349134	46311	49.026	57.059
2	31.961	2442433	34852	50.974	42.941

6c (89% ee) in Table 2, entry 3

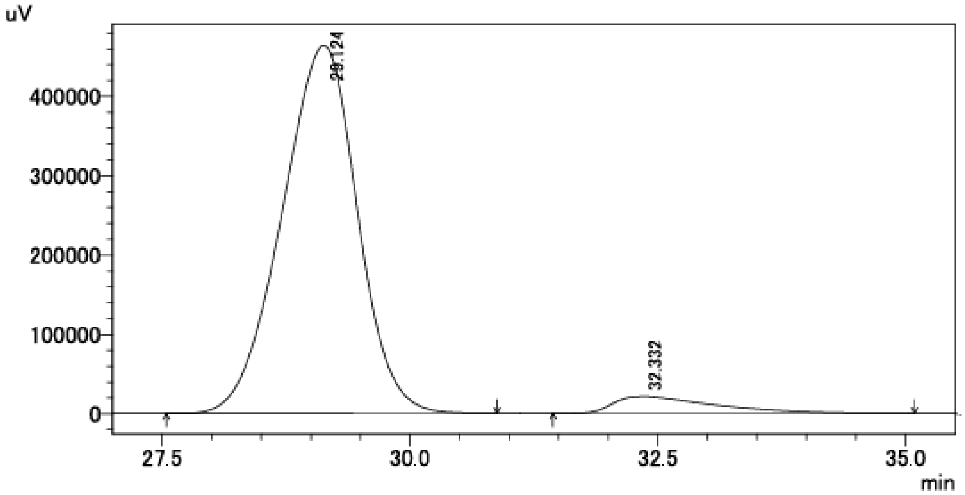

1 PDA Multi 1/254nm 4nm

<Peak Report>

Peak Table

PDA Ch1 254nm 4nm

Peak#	retention time	area	height	area%	height%
1	29.124	24547105	463907	94.314	95.622
2	32.332	1479816	21241	5.686	4.378

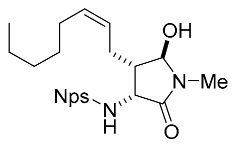

6d

(COSMOSIL CHiRAL 3A; hexane/*i*-PrOH 90:10; 1.0 mL/min; 254 nm)

racemate

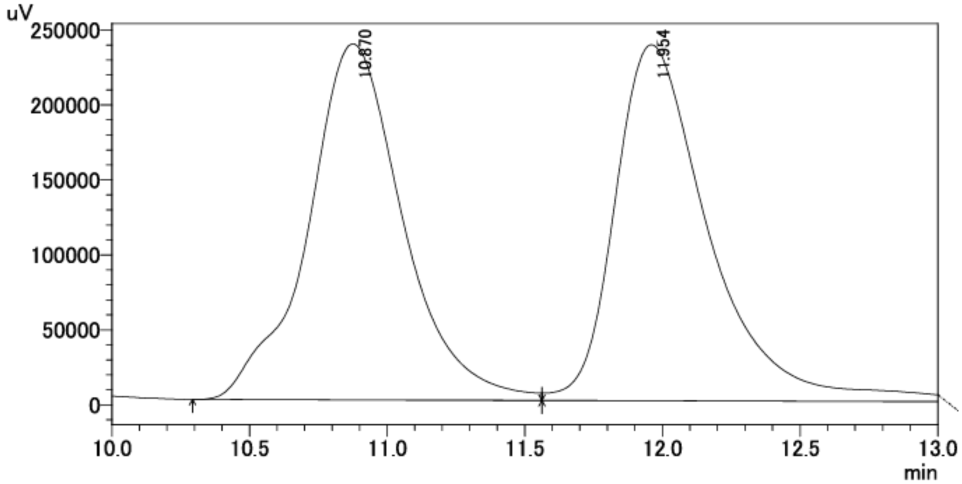

1 PDA Multi 1/254nm 4nm

<Peak Report>

Peak Table

PDA Ch1 254nm 4nm

Peak#	retention time	area	height	area%	height%
1	10.870	5726371	237455	49.839	49.991
2	11.954	5763483	237545	50.161	50.009
[illegible]		[illegible]	[illegible]	[illegible]	[illegible]

6d (81% ee) in Table 2, entry 4

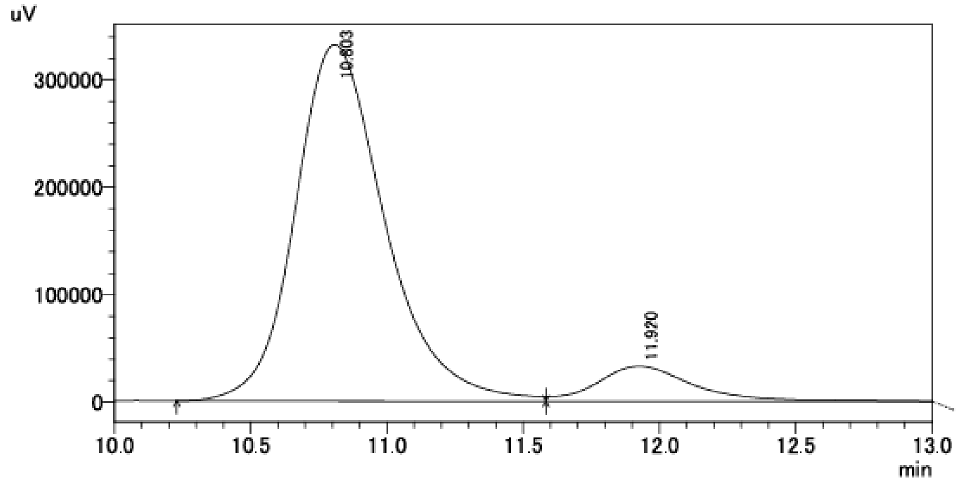

1 PDA Multi 1/254nm 4nm

<Peak Report>

Peak Table

PDA Ch1 254nm 4nm

Peak#	retention time	area	height	area%	height%
1	10.803	7609466	331876	90.310	91.023
2	11.920	816465	32729	9.690	8.977
[illegible]		[illegible]	[illegible]	[illegible]	[illegible]

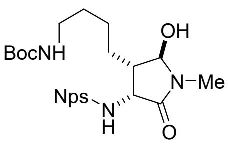

6e

(COSMOSIL CHiRAL 3C; hexane/*i*-PrOH 80:20; 1.0 mL/min; 254 nm)

racemate

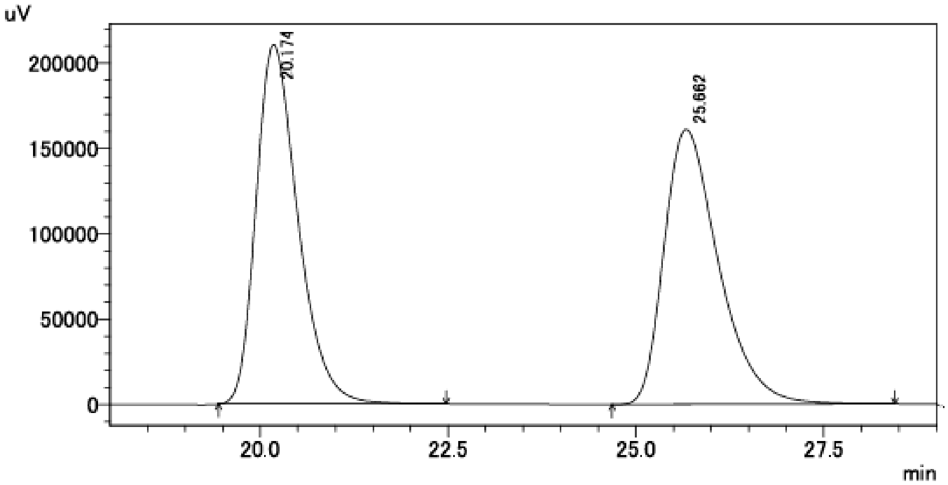

1 PDA Multi 1/254nm 4nm

<Peak Report>

Peak Table

PDA Ch1 254nm 4nm

Peak#	retention time	area	height	area%	height%
1	20.174	8167693	210414	50.001	56.642
2	25.662	8167319	161067	49.999	43.358

6e (89% ee) in Table 2, entry 5

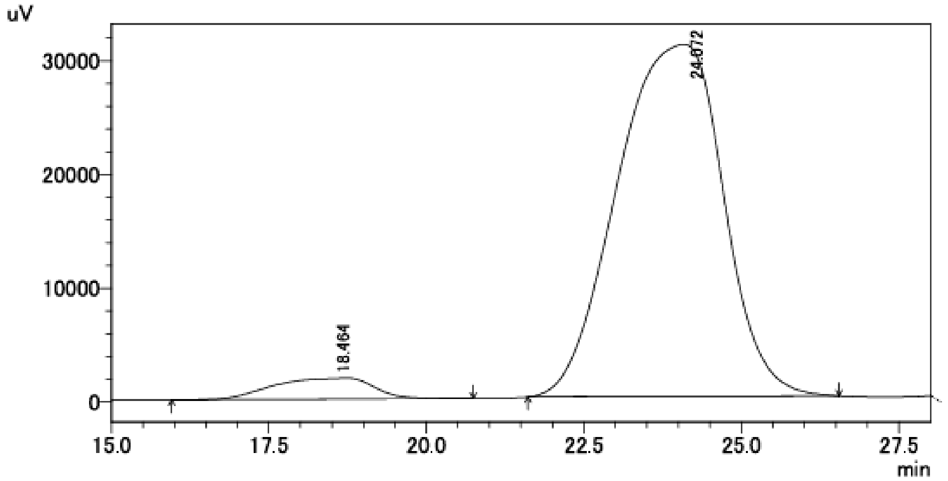

1 PDA Multi 1/254nm 4nm

<Peak Report>

Peak Table

PDA Ch1 254nm 4nm

Peak#	retention time	area	height	area%	height%
1	18.464	204082	1814	5.329	5.535
2	24.072	3625541	30955	94.671	94.465

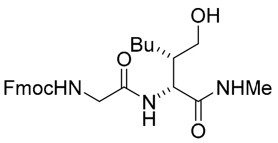

12

(Daicel chiralpak ADH; hexane/*i*-PrOH 80:20; 1.0 mL/min; 254 nm)

racemate

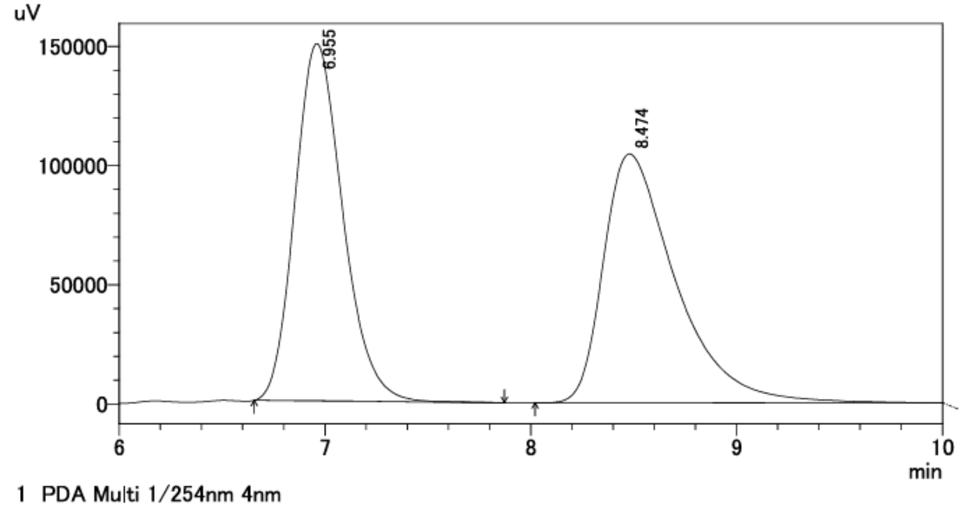

<Peak Report>

Peak Table

PDA Ch1 254nm 4nm

Peak#	retention time	area	height	area%	height%
1	6.955	2430365	149856	48.721	58.907
2	8.474	2557961	104540	51.279	41.093

12 (92% ee) in Scheme 3

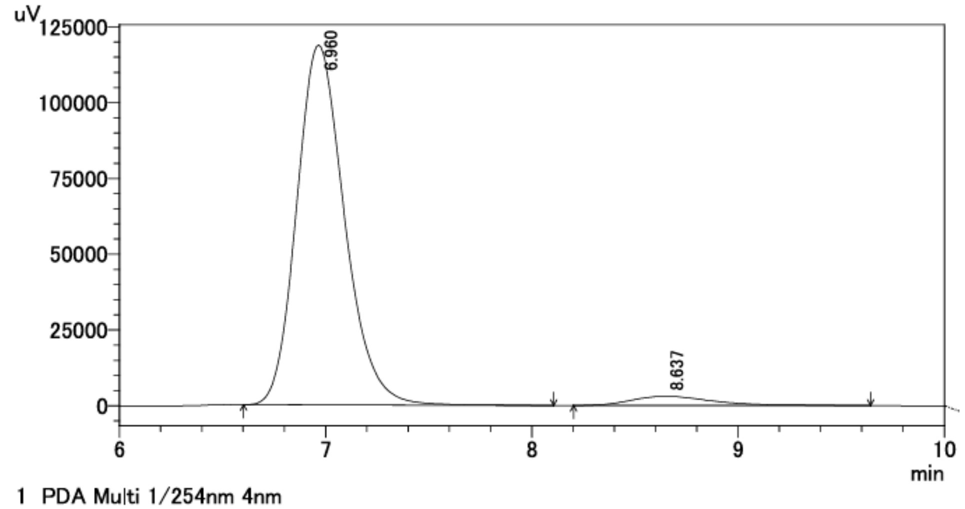

<Peak Report>

Peak Table

PDA Ch1 254nm 4nm

Peak#	retention time	area	height	area%	height%
1	6.960	1920401	118816	95.760	97.469
2	8.637	85031	3085	4.240	2.531
